# Satisfaction with life in relation to sleep health among a nationally representative sample of U.S. adults

**DOI:** 10.1038/s41598-026-41318-4

**Published:** 2026-05-15

**Authors:** Bethany T. Ogbenna, Symielle A. Gaston, Wensu Zhou, Christopher Payne, W. Braxton Jackson II, Chandra L. Jackson

**Affiliations:** 1 Epidemiology Branch, National Institute of Environmental Health Sciences, National Institutes of Health, 111 TW Alexander Drive, Room A327, Research Triangle Park, Research Triangle Park, 27709 NC USA; 2 DLH, LLC, Bethesda, MD USA

**Keywords:** Personal satisfaction, Sleep, Age groups, Sex, Population groups, US, Diseases, Health care, Medical research, Psychology, Psychology, Risk factors

## Abstract

To investigate associations between life satisfaction and sleep health among adults in the United States, we analyzed cross-sectional, nationally-representative data from the 2022 National Health Interview Survey. Life satisfaction was dichotomized as ‘very satisfied/satisfied’ vs. ‘dissatisfied/very dissatisfied.’ Sleep duration was defined as ‘recommended’ vs. ‘short’ (≥ 7 vs.<7 h), infrequent insomnia symptoms as difficulty falling/staying asleep: ‘yes’ [never/some days for both] vs. ‘no’ [most days/every day to either], and restorative sleep as feeling well rested in the past 30 days: ‘yes’ [most days/every day] vs. ‘no’ [never/some days]. Using survey-weighted Poisson regression with robust variance adjusting for confounders, we estimated prevalence ratios (aPR) and 95% confidence intervals (CI) overall and by age, sex, race, and ethnicity to test for effect modification. Among 25,090 adults (mean age of 48.1 ± 0.17 years; 54% women), 96.0% reported life satisfaction with comparable prevalence across age (18–30 years [96.6%], 31–49 years [96.4%], and ≥ 50 years [95.0%]) and among men [95.6%] along with women [95.6%]. Prevalence by race and ethnicity ranged from 93.9% [non-Hispanic (NH)-multiracial/other] to 98.0% [NH-Asian]. Life satisfaction vs. dissatisfaction was associated with recommended sleep duration (aPR:1.14 [95% CI:1.07–1.21]), infrequent insomnia symptoms (aPR:1.25 [95% CI:1.16–1.33]), and restorative sleep (aPR:1.61 [95% CI:1.45–1.79]) even after further adjustment. Age, sex, race, and ethnicity did not modify associations between life satisfaction and sleep. Life satisfaction was associated with recommended sleep duration, infrequent insomnia symptoms, and restorative sleep. Pathways underlying the life satisfaction-sleep relationship should be identified to inform interventions.

**Supplementary Information:**

The online version contains supplementary material available at 10.1038/s41598-026-41318-4.

## Introduction

The National Sleep Foundation (NSF) conducted a ‘Sleep in America^®^’ poll and found adults in the United States (U.S.) with good vs. poor sleep were significantly more likely to report flourishing or an optimal state of well-being^1^. Good sleep was defined as meeting NSF sleep duration recommendations, reporting sleep satisfaction, and having no trouble falling asleep^1^. With a critical role in shaping overall well-being, sleep is increasingly recognized as a pillar of mental, emotional, and physical health. Sleep, for instance, influences key biological processes by modulating activity within the hypothalamic-pituitary-adrenal (HPA) axis, reducing cortisol secretion, and promoting synaptic plasticity—mechanisms that collectively support emotional regulation and effective stress management^2–5^.

Satisfaction with life, distinct from flourishing as a dynamic process of realizing potential across domains, is a different but related indicator of well-being. It reflects an individuals’ overall subjective emotional and cognitive assessment of their life along with the degree to which they are content with their life circumstances^6,7^. Life satisfaction is theorized to encompass several domains including, a suitable housing and living environment; quality personal and community relationships; and time to engage in enjoyable and/or meaningful activities (including spiritual). It also reflects socioeconomic viability, such as economic stability, job satisfaction, and sufficient work-life integration^8^.

Although life satisfaction has been linked to positive health outcomes, its relationship with sleep health remains understudied. The limited published research suggests a bidirectional association between life satisfaction and sleep. Higher life satisfaction may promote favorable sleep through reduced stress, healthier habits (e.g., physical activity; nutrition), and a more positive outlook, while adequate sleep duration, quality, and timing may enhance life satisfaction by supporting emotional regulation, mitigating anxiety and negative mood, and promoting both physical health and cognitive function^9–13^. With few studies conducted in the U.S., mainly international studies have reported associations between higher life satisfaction and longer sleep duration or better sleep quality^11,14–17^. For example, studies in Germany and China found life satisfaction was associated with fewer sleep complaints. In contrast, research in Finland and the Czech Republic emphasized the role of sleep quality^15–18^. However, these studies often focus on middle-aged or older adults, and have limited generalizability to younger, more heterogenous populations like the U.S.^18–20^.

Understanding the association between life satisfaction and sleep is important among U.S. adults with varying social characteristics for several reasons. There is known variation in access to health-promoting resources and differential life experiences across sociodemographic groups^21,22^. For instance, life satisfaction may increase with age but can decline in later life due to chronic conditions or social isolation^23^. While women often report higher life satisfaction than men, they also experience a greater burden of caregiving roles, depression and insomnia symptoms, and financial stress. These patterns are partly attributable to a higher likelihood of having a low-income or living in poverty^24^. Furthermore, racial and ethnic differences are well-documented. Non-Hispanic (NH)-Black and Hispanic adults report lower life satisfaction than NH-White adults, potentially due to, on average, lower access to high-quality education and employment opportunities that can lead to lower socioeconomic status and psychological stress^25,26^. Moreover, short sleep duration is more prevalent among certain racial and ethnic groups, including NH-Black and NH-Pacific Islander, compared to NH-White adults^27,28^. However, no prior studies, to our knowledge, have assessed the relationship between life satisfaction and sleep health across a large, nationally-representative sample of the U.S. population. Variation by age, sex, race, and ethnicity have also not been studied.

To address these gaps, we determined: (1) the prevalence of life satisfaction overall and by age, sex, and race along with ethnicity; (2) cross-sectional associations between life satisfaction and sleep health overall and within these groups; and (3) differences in sleep health among racial and ethnic groups reporting life satisfaction compared to NH-White adults reporting life satisfaction. We further assessed these associations within age- and sex-specific groups (e.g., comparing Hispanic/Latino adults aged 18–30 years reporting satisfaction or dissatisfaction with NH-White adults reporting satisfaction). We hypothesized that life satisfaction would be more prevalent among younger versus older adults, women versus men, and NH-White versus other racial and ethnic groups. We also hypothesized that life satisfaction would be associated with recommended sleep duration, infrequent insomnia symptoms, and restorative sleep, with stronger associations among younger adults, women, and NH-White adults relative to their counterparts. Finally, we expected Hispanic/Latino, NH-American Indian/Alaska Native, NH-Asian, NH-Black, and NH-multiracial/Other adults reporting life satisfaction to have lower prevalence of recommended sleep, infrequent insomnia symptoms, and restorative sleep compared to NH-White adults with life satisfaction. We further hypothesized that even lower prevalence among these groups when reporting life dissatisfaction.

## Methods

### Data source: The National Health Interview Survey

The National Health Interview Survey (NHIS) is a nationally representative household interview survey of the non-institutionalized U.S. population. The NHIS uses a complex, multistage probability sample design that incorporates stratification, clustering, and oversampling of certain subgroups (e.g., older adults). The survey is conducted annually via face-to-face interviews with telephone follow-ups and self-administered questionnaires, and collects information on sociodemographic, health behaviors, chronic conditions, and healthcare access and utilization. Additional details on the sampling design and study description were previously described^29,30^. Informed consent was obtained from all participants. The Institutional Review Board (IRB) of the National Institute of Environmental Health Sciences (NIEHS) determined that approval was not required for the use of publicly available, de-identified secondary data analysis.

### Study population

Using the Integrated Public Use Microdata Series (IPUMS), we obtained cross-sectional data from the 2022 NHIS survey composed of 35,115 adults^31^. Participants were excluded if missing data for the following: all sleep measures (*n* = 1,044), life satisfaction (*n* = 58), age (*n* = 56), sex (*n* = 3), self-identified race and ethnicity (*n* = 0), or potential confounders (*n* = 1,400) yielding a total of 2,561 excluded from the sample (7.3%). The final analytic sample included 25,090 adults (Supplemental Fig. 1). Participants excluded from the analytic sample were more likely to: be women, identify as NH-Black; attain ≤ high school degree; live in the South; and report lifetime abstinence from alcohol, physically inactivity, fair/poor health, and recommended sleep duration. Excluded participants were less likely to be employed, married/living with a partner, and ever have depression (Supplemental Table 1).

### Exposure assessment: Satisfaction with life

Satisfaction with life was measured via self-reported questionnaire by asking participants, “In general, how satisfied are you with your life? Would you say very satisfied, satisfied, dissatisfied, or very dissatisfied?” Responses were dichotomized as ‘very satisfied/satisfied’ vs. ‘dissatisfied/very dissatisfied.

### Outcome assessment: Sleep duration and disturbances

Participants were asked to report their sleep duration by responding to the question, “On average, how many hours of sleep do you get in a 24-hour period?” Responses were recorded as whole numbers, with durations of ≥ 30 min rounded up to the nearest hour and durations < 30 min rounded down to nearest hour. Responses were categorized as recommended (≥ 7 h) vs. short (< 7 h) sleep duration based on the guidelines from the American Academy of Sleep Medicine and Sleep Research Society^32^. Infrequent insomnia symptoms were measured by participants’ responding to two questions: (1) “During the past 30 days, how often did you have trouble falling asleep?” and (2) “How often did you have trouble staying asleep?” Responses options included “never”, “some days”, “most days” or “every day. Infrequent insomnia symptoms were dichotomized as ‘yes’ if participants responded “never or “some days” to both questions vs. ’no’ if participants responded “most days” or “every day” to either. Restorative sleep was measured using the question, “During the past 30 days, how often did you wake up feeling well-rested?” with responses dichotomized as ‘yes’ [most days/every day] vs. ‘no’ [never/some days].

### Potential confounders

Potential confounders were identified a priori from prior literature and included the following: age (years), sex (man, woman), self-reported race and ethnicity (Hispanic/Latino, NH-American Indian/Alaska Native, NH-Asian, NH-Black/African American, NH-multiracial or other group, NH-White), educational attainment (≤ high school, some college, ≥ college), employment status (employed - currently working, employed - not currently working, not employed - homemaker, not employed - going to school, not employed - retired, not employed - not able to work, not employed - looking for work, not employed - other), and marital status (divorced/widowed, single/no live-in partner, married/living with partner/co-habituating), region of residence (Northeast, Midwest, South, West), cigarette smoking status (never/quit > 12 months prior to interview, former, current), alcohol consumption (current, former, lifetime abstainer), leisure-time physical activity (inactive, insufficiently active, sufficiently active), depression (yes, no), and body mass index (BMI; underweight [< 18.5 kg/m^2^], recommended [18.5–24.9 kg/m^2^], overweight [25–29.9 kg/m^2^], obesity [≥ 30 kg/m^2^]).

### Potential effect measure modifiers

We investigated the following potential effect modifiers: age category (18–30, 31–49, ≥ 50 years), sex, as well as combined race and ethnicity based on prior literature suggesting these sociodemographic factors may modify satisfaction with life^23,25,33^.

### Statistical analysis

Descriptive statistics were estimated overall and by life satisfaction (satisfied vs. dissatisfied). We reported sample weighted means (+/- standard errors) for age and age-standardized (consistent with the 2020 U.S. Census population) sample weighted proportions for categorical variables. The NHIS sampling weights used in these analyses account for the inverse probability of selection to reflect the complex survey design and correct for non-response. Poisson regression with robust standard errors^34,35^ was used to estimate adjusted prevalence ratios and 95% confidence intervals (aPR [95%CI]) for associations between life satisfaction and sleep health adjusting for potential confounders in the overall population and stratified by potential modifiers (i.e., age, sex, race and ethnicity), separately. Model 1 was adjusted for age, sex, race and ethnicity (when models were not stratified by these variables), marital status, educational attainment, employment status, and general health status. Model 2 was further adjusted for potential mediators: BMI, leisure-time physical activity, smoking status, alcohol consumption, and depression (Supplemental Table 2). To test for effect modification, cross-product interaction terms (e.g., life satisfaction*age, life satisfaction*sex, and life satisfaction*race/ethnicity) were included in the overall model containing Model 2 covariates. All racial and ethnic groups were included in analyses, however, due to heterogeneity, impactful results are interpreted in the text based on either magnitude of point estimates or significant alpha testing. All analyses were performed using estimation and post-estimation commands for survey data in Stata, Version 15.1 (Statacorp, College Station, Texas), and a two-sided p-value of 0.05 was used to determine statistical significance. All results in text, unless otherwise stated, are reported from fully adjusted models (Model 2).

## Results

### Study population Characteristics

Among participants (*N* = 25,090; 54.0% women), the mean age (SE) was 48 (0.2) years (Table 1). Overall, satisfaction with life was prevalent (95.6%) and comparable between men (95.6%) and women (95.6%) (Table 1) as well as across age groups: 18–30 years (96.6%), 31–49 years (96.4%), and ≥ 50 years (95.0%) (Supplemental Table 3). Satisfaction with life varied by race and ethnicity among Hispanic/Latine (96.5%), NH-American Indian/Alaska Native (93.0%), NH-Asian (98.0%), NH-Black (95.3%), NH-multiracial/Other (93.9%), and NH-White (95.4%) adults (see Supplemental Table 4). Among participants satisfied with life, there was a higher proportion of adults completing ≥ college (33.8% vs. 19.2% dissatisfied), being employed (64.6% vs. 41.0% dissatisfied), being married (60.2% vs. 34.6% dissatisfied), living in the southern U.S. region (38.0% vs. 35.4% dissatisfied), never smoking (87.8% vs. 73.9% dissatisfied), consuming alcohol (70.2% vs. 64.4% dissatisfied), being sufficiently physically active (48.5% vs. 32.3% dissatisfied), having a BMI within the recommended range (31.3% vs. 28.2% dissatisfied), and reporting good/very good/excellent general health (87.7% vs. 48.2% dissatisfied). Adults who were satisfied with life had a higher prevalence of favorable sleep outcomes compared to adults who were dissatisfied: 70.3% vs. 54.6% for recommended sleep duration, 77.8% vs. 49.6% for infrequent insomnia symptoms, and 58.3% vs. 25.7% for restorative sleep.

**Table 1:** Study population characteristics, overall and by sex, National Health Interview Survey, 2022, (*N* = 25,090).

	Total *n* = 25,090 (100%)			Men *n* = 11,532 (46.0%)			Women *n* = 13,558 (54.0%)		
		Life satisfaction ^a^			Life satisfaction ^a^			Life satisfaction ^a^	
	All *n* = 25,090 (100%)	Yes *n* = 23,997 (95.6%)	No *n* = 1,093 (4.4%)	All *n* = 11,532 (100%)	Yes *n* = 11,030 (95.6%)	No *n* = 502 (4.4%)	All *n* = 13,558 (100%)	Yes *n* = 12,967 (95.6%)	No *n* = 591 (4.4%)
**Sociodemographic Characteristics**									
*Age (years), mean (SE)*	48.1 (0.17)	48.0 (0.17)	50.5 (0.75)	47.4 (0.21)	47.4 (0.22)	48.2 (1.1)	48.7 (0.21)	48.5 (0.21)	52.8 (0.94)
18–30 years	21.8	21.9	20.0	22.5	22.4	25.0	21.1	21.4	14.9
31–49 years	31.5	31.7	26.1	31.9	32.2	25.7	31.0	31.2	26.4
≥ 50 years	46.7	46.4	53.9	45.6	45.4	49.3	47.9	47.4	58.7
*Race and ethnicity*									
Hispanic/Latine	16.9	17.1	13.6	16.9	17.0	13.5	17.0	17.2	13.6
NH-American Indian/Alaska Native	0.8	0.8	1.0	0.6	0.6	1.4	0.9	1.0	0.5
NH-Asian	6.1	6.3	2.7	5.7	5.8	2.5	6.5	6.7	2.7
NH-Black/African American	11.2	11.1	13.5	10.1	10.0	13.4	12.2	12.2	13.5
NH-multiracial or other group^b^	2.0	2.0	3.4	2.1	2.1	3.7	1.9	1.9	3.3
NH-White	62.9	62.8	65.9	64.6	64.5	65.6	61.3	61.1	66.5
*Educational Attainment*									
≤ High School	37.2	36.8	47.3	39.1	38.7	50.5	35.1	34.8	42.9
Some college	29.6	29.5	33.6	28.3	28.1	31.9	30.9	30.8	36.1
≥ College	33.2	33.8	19.2	32.6	33.2	17.7	33.9	34.4	21.0
*Employment status*									
Employed, currently working	63.7	64.6	41.0	68.6	69.7	43.1	58.9	59.7	38.9
Employed, not currently working	0.6	0.6	0.9	0.6	0.6	0.7	0.6	0.6	1.0
Not employed, Homemaker	4.7	4.7	4.7	0.9	0.8	1.8	8.6	8.6	8.4
Not employed, Going to school	2.3	2.4	1.6	2.2	2.2	2.5	2.5	2.6	0.4
Not employed, Retired	18.9	19.0	16.8	18.0	18.1	16.0	19.7	19.8	17.5
Not employed, not able to work	6.0	5.1	25.0	5.7	4.9	23.8	6.2	5.3	26.0
Not employed, looking for work	2.2	2.0	6.3	2.5	2.3	8.0	1.9	1.8	4.5
Not employed, Other	1.6	1.5	3.8	1.5	1.4	4.1	1.6	1.6	3.3
*Marital status*									
Divorced/widowed	17.2	16.7	28.0	13.2	12.8	22.4	20.8	20.2	33.4
Single/no live-in partner	23.6	23.1	37.4	25.3	24.5	42.7	22.0	21.7	30.5
Married/living with partner/cohabiting	59.2	60.2	34.6	61.5	62.7	34.9	57.2	58.1	36.1
*Region of residence*									
Northeast	17.2	17.2	17.8	17.3	17.3	17.6	17.1	17.1	17.7
Midwest	21.0	21.0	22.1	21.4	21.4	21.2	20.7	20.6	22.7
South	37.9	38.0	35.4	37.3	37.4	35.1	38.6	38.7	36.4
West	23.8	23.8	24.7	24.0	24.0	26.1	23.7	23.7	23.1
**Health Behaviors**									
*Smoking status*									
Never/quit > 12 months prior	87.3	87.8	73.9	85.5	86.1	73.2	89.0	89.5	74.6
Former/quit ≤ 12 months ago	1.2	1.2	2.4	1.4	1.4	3.2	0.9	0.9	1.5
Current	11.5	11.1	23.7	13.0	12.6	23.5	10.1	9.6	23.9
*Alcohol consumption*									
Current (≥ 1 drink past year)	69.9	70.2	64.4	72.8	73.0	67.6	67.3	67.6	62.4
Former (no drinks past year)	17.0	16.7	23.8	17.2	17.0	22.5	16.8	16.5	24.4
Lifetime abstinence (< 12 drinks in life)	13.0	13.1	11.7	10.0	10.0	9.9	15.9	16.0	13.2
*Leisure-time physical activity* ^*c*^									
Inactive	26.5	25.8	44.3	24.7	23.9	42.6	28.3	27.5	46.5
Insufficiently active	25.6	25.7	23.4	22.7	22.8	20.5	28.5	28.6	26.4
Sufficiently active	47.8	48.5	32.3	52.6	53.3	36.9	43.2	43.9	27.0
*Usual sleep duration*									
Short (< 7 h)	30.3	29.7	45.4	29.9	29.3	44.0	30.6	30.0	46.9
Recommended (≥ 7 h)	69.7	70.3	54.6	70.1	70.7	56.0	69.4	70.0	53.1
*Infrequent Insomnia symptoms (yes) * ^*d*^	76.7	77.8	49.6	80.1	81.2	53.0	73.4	74.5	46.1
*Restorative sleep (yes)* ^*e*^	57.0	58.3	25.7	60.6	62.1	27.0	53.3	54.5	24.3
**Clinical Characteristics**									
*Ever had depression (yes)* ^*f*^	18.5	16.9	55.2	13.2	11.6	49.0	23.6	22.0	62.2
*Body mass index category*									
Underweight (< 18.5 kg/m^2^)	1.7	1.6	2.8	1.1	1.1	1.3	2.2	2.1	4.3
Recommended (18.5-<25 kg/m^2^)	31.2	31.3	28.2	27.0	26.9	29.2	35.3	35.6	26.7
Overweight (25-<30 kg/m^2^)	33.7	34.0	27.0	38.8	39.0	34.2	28.8	29.2	19.4
Obesity (≥ 30 kg/m^2^)	33.4	33.0	42.0	33.1	33.0	35.4	33.7	33.1	49.6
*General health status*									
Fair/poor	13.9	12.3	51.8	13.6	12.0	50.4	14.3	12.6	53.8
Good/very good/excellent	86.1	87.7	48.2	86.4	88.0	49.6	85.7	87.4	46.2

Abbreviations: SE (standard error), NH (non-Hispanic).

Note: Data are presented as column percentages or means and standard errors. Percentages may not sum to 100 due to missing and/or rounding. All estimates are weighted for the survey’s complex sampling design. All estimates are age-standardized to the U.S. 2020 population, except for age.

^a^Life satisfaction was ascertained based on the questions, ‘In general, how satisfied are you with your life? Are you very satisfied, satisfied, dissatisfied, or very dissatisfied?‘. Life satisfaction was dichotomized as yes (a response of ‘satisfied’ or ‘very satisfied’) vs. no (a response of ‘very dissatisfied’ or ‘dissatisfied’).

^b^NH-Other single and multiple races and ‘Other group’ is defined as persons identifying with racial groups not explicitly listed in the standard categories.

^c^Leisure-time physical activity was defined using the 2018 Health and Human Services Physical Activity guidelines which state “recommend that adults complete at least 150 minutes to 300 minutes of moderate-intensity activity, or 75 minutes to 150 minutes of vigorous-intensity aerobic activity per week, as well as moderate or greater intensity muscle strengthening activities on two or more days a week.”.

^d^Infrequent insomnia symptoms (yes) defined as difficulty falling or staying asleep never/some days in the past 30 days.

^e^Restorative sleep (yes) defined as most days/every day waking up feeling rested in the past 30 days.

^f^Depression was defined by the 2019 Field Representative’s Manual as a major depressive disorder or as clinical depression that is a common but serious mood disorder. “It causes severe symptoms that affect how you feel, think, and handle daily activities, such as sleeping, eating, or working.”.

### Life satisfaction and sleep health in the overall population

Overall, life satisfaction versus dissatisfaction was associated with a 14% higher prevalence of recommended sleep duration (aPR: 1.14, 95% CI: 1.07, 1.21), a 25% higher prevalence of infrequent insomnia symptoms (aPR:1.25 95% CI:1.16, 1.33), and a 61% higher prevalence of restorative sleep (aPR:1.61, 95% CI:1.45, 1.79) (Table 2).

**Table 2:** Prevalence ratios for associations between life satisfaction and sleep, overall and stratified by race along with ethnicity and age, National Health Interview Survey, 2022, (*N* = 25,090).

	Recommended Sleep Duration (≥ 7 h) vs. Short (< 7 h) ^a^		Infrequent insomnia symptoms (yes vs. no) ^b^		Restorative Sleep (yes vs. no) ^a, c^	
	Model 1 ^d^	Model 2 ^d^	Model 1 ^d^	Model 2 ^d^	Model 1 ^d^	Model 2 ^d^
	Prevalence Ratio (95% Confidence Interval) for Associations with Life satisfaction ^e^ (satisfied vs. dissatisfied)					
**Overall (*n*** **= 25,090)**	1.18	1.14	1.36	1.25	1.83	1.61
	(1.11–1.26)	(1.07–1.21)	(1.27–1.45)	(1.16–1.33)	(1.65–2.04)	(1.45–1.79)
18–30 years (*n* = 3,639)	1.31	1.24	1.41	1.26	2.41	2.04
	(1.10–1.56)	(1.04–1.47)	(1.16–1.70)	(1.04–1.53)	(1.61–3.59)	(1.38–3.02)
31–49 years (*n* = 7,198)	1.20	1.14	1.39	1.28	1.90	1.68
	(1.04–1.38)	(0.99–1.31)	(1.21–1.60)	(1.12–1.46)	(1.48–2.43)	(1.31–2.15)
≥ 50 Years (*n* = 14,253)	1.11	1.09	1.31	1.23	1.62	1.46
	(1.04–1.20)	(1.02–1.17)	(1.20–1.43)	(1.13–1.34)	(1.43–1.84)	(1.29–1.64)
**Hispanic/Latino (*n*** **= 3,511)**^**f**^	1.38	1.27	1.72	1.54	2.21	1.91
	(1.09–1.74)	(1.01–1.60)	(1.35–2.20)	(1.22–1.95)	(1.53–3.19)	(1.34–2.70)
18–30 years (*n* = 851)	1.49	1.33	2.26	2.02	3.46	3.02
	(0.96–2.31)	(0.86–2.06)	(1.29–3.95)	(1.17–3.49)	(1.45–8.23)	(1.30–7.00)
31–49 years (*n* = 1,335)	2.11	1.90	1.61	1.40	1.86	1.47
	(1.19–3.72)	(1.07–3.37)	(1.11–2.34)	(1.00–1.96)	(1.01–3.44)	(0.82–2.65)
≥ 50 Years (*n* = 1,325)	1.08	1.06	1.47	1.37	1.88	1.77
	(0.83–1.41)	(0.83–1.37)	(1.08–2.01)	(1.03–1.83)	(1.10–3.21)	(1.09–2.88)
**NH-American Indian/Alaska Native (*n*** **= 172)**	1.81	1.59	2.08	1.29	0.95	0.62
	(0.90–3.62)	(0.92–2.76)	(0.71–6.08)	(0.62–2.69)	(0.43–2.12)	(0.25–1.54)
18–30 years (*n* = 35)	1.76	0.23	NE	NE	NE	NE
	(0.23–13.7)	(0.05–1.05)				
31–49 years (*n* = 54)	NE	NE	NE	NE	NE	NE
≥ 50 Years (*n* = 83)	3.17	3.44	1.55	1.71	1.28	1.39
	(1.02–9.90)	(1.18–10.0)	(0.80–3.00)	(1.14–2.59)	(0.64–2.59)	(0.63–3.05)
**NH-Asian (** ***n*** ** = 1,531)**	1.06	1.04	1.27	1.19	1.40	1.25
	(0.78–1.43)	(0.77–1.42)	(0.96–1.68)	(0.90–1.56)	(0.83–2.36)	(0.76–2.06)
18–30 years (*n* = 272)	3.28	2.67	1.19	1.18	0.91	0.74
	(0.39–27.8)	(0.33–21.6)	(0.66–2.13)	(0.59–2.38)	(0.54–1.52)	(0.33–1.66)
31–49 years (*n* = 638)	0.89	0.85	1.21	1.15	1.52	1.43
	(0.50–1.58)	(0.47–1.54)	(0.65–2.26)	(0.62–2.14)	(0.50–4.62)	(0.51–4.00)
≥ 50 Years (*n* = 621)	0.95	0.98	1.23	1.15	1.50	1.35
	(0.70–1.29)	(0.71–1.34)	(0.87–1.74)	(0.84–1.57)	(0.77–2.90)	(0.75–2.44)
**NH-Black/African American (*n*** **= 2,664)**	1.41	1.33	1.33	1.25	1.71	1.53
	(1.10–1.80)	(1.05–1.69)	(1.11–1.60)	(1.05–1.49)	(1.17–2.48)	(1.08–2.17)
18–30 years (*n* = 413)	1.16	1.16	1.03	0.97	1.33	1.14
	(0.76–1.78)	(0.78–1.72)	(0.80–1.32)	(0.75–1.25)	(0.66–2.67)	(0.64–2.06)
31–49 years (*n* = 792)	1.85	NE	2.53	NE	5.45	NE
	(1.05–3.26)		(1.44–4.46)		(1.97–15.1)	
≥ 50 Years (*n* = 1,459)	1.42	1.31	1.22	1.12	1.38	1.24
	(1.02–1.99)	(0.94–1.81)	(0.99–1.51)	(0.91–1.38)	(0.96–2.00)	(0.86–1.78)
**NH-multiracial or other group (*n*** **= 456)**^**g, h**^	1.44	1.40	1.54	1.37	1.18	0.99
	(0.84–2.45)	(0.82–2.39)	(0.93–2.54)	(0.87–2.15)	(0.71–1.98)	(0.59–1.68)
18–30 years (*n* = 139)	NE	NE	NE	NE	NE	NE
31–49 years (*n* = 160)	1.31	1.33	0.81	0.78	0.84	0.81
	(0.51–3.39)	(0.44–4.00)	(0.55–1.19)	(0.55–1.12)	(0.37–1.88)	(0.37–1.78)
≥ 50 Years (*n* = 157)	0.89	0.88	1.59	1.48	0.82	0.73
	(0.55–1.45)	(0.55–1.42)	(0.69–3.63)	(0.61–3.60)	(0.44–1.53)	(0.41–1.31)
**NH-White (*n*** **= 16,756)**^**f**^	1.10	1.07	1.28	1.18	1.87	1.65
	(1.03–1.18)	(1.00–1.15)	(1.18–1.39)	(1.08–1.28)	(1.64–2.14)	(1.45–1.87)
18–30 years (*n* = 1,929)	1.19	1.14	1.30	1.15	3.26	2.72
	(0.97–1.46)	(0.93–1.39)	(1.02–1.68)	(0.88–1.49)	(1.72–6.18)	(1.45–5.11)
31–49 years (*n* = 4,219)	1.03	0.98	1.22	1.11	1.80	1.57
	(0.89–1.19)	(0.85–1.13)	(1.04–1.42)	(0.96–1.30)	(1.32–2.45)	(1.15–2.12)
50 Years (*n* = 10,608)	1.09	1.07	1.30	1.22	1.66	1.49
	(1.01–1.19)	(0.99–1.16)	(1.17–1.44)	(1.10–1.35)	(1.44–1.92)	(1.30–1.72)

Abbreviations: NH (non-Hispanic), NE (not able to estimate).

^a^Significant interaction between age and life satisfaction on sleep outcome: recommended sleep duration (p *interaction* = 0.0251) and restorative sleep (p *interaction* = 0.0130) in the overall population.

^b^Infrequent insomnia symptoms (yes) defined as difficulty falling or staying asleep never/some days in past 30 days.

^c^Restorative sleep (yes) defined as most days/every day waking up feeling rested in the past 30 days.

^d^Model 1 is adjusted for age (years) when not stratified by age, sex (man, woman), marital status (divorced/widowed, single/no live-in partner, married/living with partner/co-habituating), educational attainment (≤ high school, some college, ≥ college), employment status (employed - currently working, employed - not currently working, not employed - homemaker, not employed - going to school, not employed - retired, not employed - not able to work, not employed - looking for work, not employed - other), and general health status (fair/poor, good/very good/excellent). Model 2 is adjusted for covariates in Model 1 and BMI (underweight (< 18.5 kg/m), recommended (18.5–24.9 kg/m), overweight (25–29.9 kg/m), obesity (≥ 30 kg/m)), leisure-time physical activity (inactive, insufficiently active, sufficiently active), smoking status (never/quit > 12 months prior to interview, former, current), alcohol consumption (current, former, lifetime abstainer), and depression (yes, no). Models in the overall sample are additionally adjusted for race and ethnicity (Hispanic/Latino, NH-American Indian/Alaska Native, NH-Asian, NH-Black/African American, NH-multiracial or other group, NH-White).

^e^Life satisfaction was ascertained based on the questions, ‘In general, how satisfied are you with your life? Are you very satisfied, satisfied, dissatisfied, or very dissatisfied?‘. Life satisfaction was dichotomized as yes (a response of ‘satisfied’ or ‘very satisfied’) vs. no (a response of ‘very dissatisfied’ or ‘dissatisfied’).

^f^Significant interaction between age and life satisfaction on restorative sleep within race and ethnicity group: Hispanic adults (p interaction = 0.0494) and NH-White (p interaction = 0.0210).

^g^Significant interaction between age and life satisfaction on sleep duration within NH-Multiracial or other group (p interaction = 0.0029).

^h^Significant interaction between age and life satisfaction on infrequent insomnia symptoms within NH-Multiracial or other group (p interaction = 0.0306).

### Life satisfaction and sleep health by age

Potential evidence of effect modification by age was observed for the associations between life satisfaction and both recommended sleep duration and restorative sleep based on p-values for cross-product interaction

 terms, however; confidence intervals for age-specific estimates largely overlapped. For instance, magnitudes of associations with recommended sleep duration were largest among adults 18–30 years 

(aPR_18−30 years_:1.24, 95% CI:1.04,1.47), followed by adults 31–49 years 

(aPR_31−49 years_:1.14, 95% CI:0.99,1.31), and adults ≥ 50 years (aPR_≥ 50 years_:1.09, 95% CI:1.02, 1.17; *p*_life satisfaction*age_ = 0.025) 

(see Table 2). 

This pattern was similar for associations between life satisfaction and restorative sleep: adults 

18–30 years (aPR_18−30 years_ :2.04, 95% CI:1.38, 3.02) followed by adults 31–49 years (aPR_31−49 years_ :1.68, 95% 

CI:1.31,2.15), and adults ≥ 50 years (aPR_≥ 50 years_ :1.46, 95% CI:1.29, 1.64; *p*_life satisfaction*age_ = 0.013).

### Life satisfaction and sleep health by sex, and by race and ethnicity

Neither sex (Table 3) nor race and ethnicity (Table 2) modified the associations between life satisfaction and sleep health dimensions among the overall population (*p*_life satisfaction*race and ethnicity_ > 0.05).

**Table 3:** Prevalence ratios for associations between life satisfaction and sleep, overall and stratified by race along with ethnicity and sex, National Health Interview Survey, 2022, (*N* = 25,090).

	Recommended Sleep Duration (≥ 7 h) vs. Short (< 7 h)		Infrequent insomnia symptoms (yes vs. no) ^a^		Restorative Sleep (yes vs. no) ^b^	
	Model 1 ^c^	Model 2 ^c^	Model 1 ^c^	Model 2 ^c^	Model 1 ^c^	Model 2 ^c^
	Prevalence Ratio (95% Confidence Interval) for Associations with Life satisfaction ^d^ (satisfied vs. dissatisfied)					
**Overall (*n*** **= 25,090)**	1.18	1.14	1.36	1.25	1.83	1.61
	(1.11–1.26)	(1.07–1.21)	(1.27–1.45)	(1.16–1.33)	(1.65–2.04)	(1.45–1.79)
Men (*n* = 11,532)	1.19	1.14	1.35	1.25	1.95	1.71
	(1.09–1.31)	(1.04–1.25)	(1.22–1.50)	(1.13–1.38)	(1.66–2.29)	(1.46–2.00)
Women (*n* = 13,558)	1.17	1.13	1.36	1.24	1.72	1.52
	(1.07–1.28)	(1.03–1.24)	(1.22–1.51)	(1.12–1.38)	(1.48–2.00)	(1.31–1.76)
**Hispanic/Latino (*n*** **= 3,511)**	1.38	1.27	1.72	1.54	2.21	1.91
	(1.09–1.74)	(1.01–1.60)	(1.35–2.20)	(1.22–1.95)	(1.53–3.19)	(1.34–2.70)
Men (*n* = 1,610)	1.49	1.39	1.78	1.60	2.32	2.01
	(1.07–2.08)	(1.00–1.94)	(1.25–2.53)	(1.13–2.27)	(1.32–4.07)	(1.16–3.48)
Women (*n* = 1,901)	1.23	1.12	1.62	1.45	2.07	1.75
	(0.92–1.66)	(0.84–1.51)	(1.17–2.24)	(1.07–1.97)	(1.25–3.43)	(1.08–2.82)
**NH-American Indian/Alaska Native (*n*** **= 172)**	1.81	1.59	2.08	1.29	0.95	0.62
	(0.90–3.62)	(0.92–2.76)	(0.71–6.08)	(0.62–2.69)	(0.43–2.12)	(0.25–1.54)
Men (*n* = 71)	1.22	0.80	7.20	3.34	1.57	1.53
	(0.62–2.42)	(0.25–2.51)	(0.67–77.8)	(0.57–19.7)	(0.57–4.27)	(0.44–5.32)
Women (*n* = 101)	1.74	NE	0.67	0.68	0.74	NE
	(1.09–2.79)		(0.43–1.05)	(0.36–1.30)	(0.37–1.48)	
**NH-Asian (*n*** **= 1,531)**	1.06	1.04	1.27	1.19	1.40	1.25
	(0.78–1.43)	(0.77–1.42)	(0.96–1.68)	(0.90–1.56)	(0.83–2.36)	(0.76–2.06)
Men (*n* = 669)	1.12	1.14	1.29	1.17	1.54	1.31
	(0.75–1.68)	(0.77–1.69)	(0.87–1.90)	(0.76–1.79)	(0.69–3.44)	(0.58–2.92)
Women (*n* = 862)	0.91	0.89	1.21	1.17	1.26	1.16
	(0.60–1.37)	(0.59–1.34)	(0.82–1.78)	(0.83–1.64)	(0.62–2.58)	(0.59–2.29)
**NH-Black/African American (*n*** **= 2,664)**	1.41	1.33	1.33	1.25	1.71	1.53
	(1.10–1.80)	(1.05–1.69)	(1.11–1.60)	(1.05–1.49)	(1.17–2.48)	(1.08–2.17)
Men (*n* = 1,083)	1.56	1.45	1.32	1.26	1.94	1.77
	(1.09–2.24)	(1.03–2.04)	(1.04–1.67)	(1.01–1.59)	(1.10–3.43)	(1.04–3.00)
Women (*n* = 1,581)	1.26	1.20	1.35	1.24	1.48	1.28
	(0.89–1.77)	(0.86–1.69)	(1.02–1.79)	(0.94–1.64)	(0.89–2.46)	(0.80–2.05)
**NH-multiracial or other group (*n*** **= 456)**	1.44	1.40	1.54	1.37	1.18	0.99
	(0.84–2.45)	(0.82–2.39)	(0.93–2.54)	(0.87–2.15)	(0.71–1.98)	(0.59–1.68)
Men (*n* = 226)	0.92	0.86	1.57	1.42	1.14	0.98
	(0.56–1.50)	(0.54–1.38)	(0.75–3.29)	(0.75–2.72)	(0.59–2.20)	(0.56–1.74)
Women (*n* = 230)	2.92	3.13	1.52	1.28	1.39	1.14
	(1.05–8.12)	(1.17–8.39)	(0.79–2.94)	(0.73–2.22)	(0.62–3.14)	(0.45–2.89)
**NH-White (*n*** **= 16,756)**	1.10	1.07	1.28	1.18	1.87	1.65
	(1.03–1.18)	(1.00–1.15)	(1.18–1.39)	(1.08–1.28)	(1.64–2.14)	(1.45–1.87)
Men (*n* = 7,873)	1.10	1.06	1.25	1.16	1.98	1.73
	(0.99–1.21)	(0.96–1.16)	(1.12–1.40)	(1.04–1.30)	(1.64–2.41)	(1.44–2.09)
Women (*n* = 8,883)	1.12	1.09	1.31	1.20	1.78	1.57
	(1.01–1.23)	(0.98–1.20)	(1.15–1.49)	(1.05–1.37)	(1.47–2.14)	(1.30–1.90)

Abbreviations: NH (non-Hispanic), NE (not able to estimate).

^a^ Infrequent insomnia symptoms (yes) defined as difficulty falling or staying asleep never/some days in past 30 days.

^b^ Restorative sleep (yes) defined as most days/every day waking up feeling rested in the past 30 days.

^c^ Model 1 is adjusted for age (years) when not stratified by age, sex (man, woman), marital status (divorced/widowed, single/no live-in partner, married/living with partner/co-habituating), educational attainment (≤ high school, some college, ≥ college), employment status (employed - currently working, employed - not currently working, not employed - homemaker, not employed - going to school, not employed - retired, not employed - not able to work, not employed - looking for work, not employed - other), and general health status (fair/poor, good/very good/excellent). Model 2 is adjusted for covariates in Model 1 and body mass index (underweight (< 18.5 kg/m), recommended (18.5–24.9 kg/m), overweight (25–29.9 kg/m), obesity (≥ 30 kg/m)), leisure-time physical activity (inactive, insufficiently active, sufficiently active), smoking status (never/quit > 12 months prior to interview, former, current), alcohol consumption (current, former, lifetime abstainer), and depression (yes, no). Models in the overall sample are additionally adjusted for race and ethnicity (Hispanic/Latino, NH-American Indian/Alaska Native, NH-Asian, NH-Black/African American, NH-Multiracial or other group, NH-White).

^d^ Life satisfaction was ascertained based on the questions, ‘In general, how satisfied are you with your life? Are you very satisfied, satisfied, dissatisfied, or very dissatisfied?‘. Life satisfaction was dichotomized as yes (a response of ‘satisfied’ or ‘very satisfied’) vs. no (a response of ‘very dissatisfied’ or ‘dissatisfied’).

### Various interrelated life satisfaction-sleep associations with age, sex, and race along with ethnicity

*Life satisfaction and sleep health by age as well as race and ethnicity*. Despite overlapping confidence intervals across age groups, the associations between life satisfaction and restorative sleep among NH-White adults appeared weaker as age increased (aPR_18−30 years_= 2.72, 95% CI:1.45,5.11; aPR_31−49 years_=1.57, 95% CI:1.15,2.12; aPR_≥ 50 years_=1.49, 95% CI:1.30, 1.72; *p*_life satisfaction*age within NH−White adults_ = 0.021) (Table 2). Within other racial and ethnic groups, age did not modify associations with life satisfaction. (Table 2).

*Life satisfaction and sleep health by sex as well as race and ethnicity*. Within racial and ethnic groups, sex did not modify associations between life satisfaction in relation to recommended sleep duration, infrequent insomnia symptoms, and restorative sleep (Table 3).

*Life satisfaction among various racial and ethnic groups compared to NH-White adults satisfied with life, overall, by age, and by sex*. Compared to NH-White adults with life satisfaction, Hispanic/Latino adults with life satisfaction had a similar prevalence of recommended sleep duration (aPR = 0.98, 95% CI: 0.95–1.01) and restorative sleep (aPR = 0.98, 95% CI: 0.95–1.02), as well as an 8% higher prevalence of infrequent insomnia symptoms (aPR = 1.08, 95% CI: 1.06–1.11) (Table 4). In contrast, Hispanic/Latino adults reporting life dissatisfaction compared to NH-White adults reporting life satisfaction had significantly lower prevalence of all three sleep outcomes: 27% lower for recommended sleep duration (aPR = 0.73, 95% CI: 0.59–0.91), 31% lower for infrequent insomnia symptoms (aPR = 0.69, 95% CI: 0.55–0.88), and 49% lower for restorative sleep (aPR = 0.51, 95% CI: 0.36–0.72). Compared to NH-White adults with life satisfaction, NH-American Indian/Alaska Native adults with life satisfaction or with dissatisfaction were no more or less likely to report all three sleep dimensions: recommended sleep duration, infrequent insomnia symptoms, restorative sleep (Table 4). Compared to NH-White adults with life satisfaction, NH-Asian adults with life satisfaction had 6% lower prevalence of recommended sleep duration (aPR = 0.94, 95% CI: 0.91–0.98), 9% higher prevalence of infrequent insomnia symptoms (aPR = 1.09, 95% CI: 1.06–1.12), and were no more or less likely to report restorative sleep (Table 4). However, no significant associations with all three sleep dimensions were observed among NH-Asian adults reporting life dissatisfaction compared to NH-White adults reporting life satisfaction. NH-Black adults with life satisfaction had a 14% lower prevalence of recommended sleep duration (aPR = 0.86, 95% CI: 0.83–0.90) and a marginally lower prevalence of restorative sleep (aPR = 0.96, 95% CI: 0.92–1.00) compared to NH-White adults who were satisfied with life. NH-Black adults reporting life dissatisfaction had a 36% lower prevalence of recommended sleep duration (aPR = 0.64, 95% CI: 0.50–0.82), a 37% lower prevalence of restorative sleep (aPR = 0.63, 95% CI: 0.45–0.88), and were no more or less likely to report infrequent insomnia symptoms. Although NH-Multiracial and other adults reporting life satisfaction had a lower prevalence of recommended sleep duration (aPR = 0.88, 95% CI: 0.81–0.97) and NH-Multiracial and other adults with life dissatisfaction had lower prevalence of infrequent insomnia symptoms (aPR = 0.62, 95% CI: 0.39–0.99) compared to NH-White adults reporting life satisfaction, the heterogeneity within multiracial groups limits the interpretability of these associations. Associations among Hispanic/Latino, NH-American Indian/Alaska Native, NH-Asian, NH-Black, and NH-Multicultural or other groups of adults reporting satisfaction or dissatisfaction with life compared to NH-White adults who were satisfied with life across all sleep dimensions did not vary by sex. There were modest variations in associations for infrequent insomnia symptoms and restorative sleep among NH-Black adults by age (Table 4).

**Table 4:** Prevalence ratios of sleep duration and sleep disturbances among Hispanic/Latino, non-Hispanic (NH)-Asian, NH-American Indian/Alaska Native, NH-Black/African American, and NH-Other single and multiple races’ reporting satisfaction or dissatisfaction with life compared to non-Hispanic White adults reporting satisfaction with life, National Health Interview Survey, 2022, (*N* = 24,314).

	Recommended Sleep Duration (≥ 7 h) vs. Short (< 7 h)				Infrequent insomnia symptoms (yes vs. no) ^a^				Restorative Sleep (yes vs. no) ^b^			
	Model 1 ^c^		Model 2 ^c^		Model 1 ^c^		Model 2 ^c^		Model 1 ^c^		Model 2 ^c^	
	Prevalence Ratio (95% Confidence Interval) for Associations with Life satisfaction ^d^ (vs. NH-White adults satisfied with life)											
	Satisfied	Dissatisfied	Satisfied	Dissatisfied	Satisfied	Dissatisfied	Satisfied	Dissatisfied	Satisfied	Dissatisfied	Satisfied	Dissatisfied
**Hispanic/Latino, overall (** ***n*** ** = 3,511)**	0.99	0.72	0.98	0.73	1.12	0.65	1.08	0.69	1.01	0.47	0.98	0.51
	(0.96–1.02)	(0.58–0.90)	(0.95–1.01)	(0.59–0.91)	(1.10–1.14)	(0.51–0.84)	(1.06–1.11)	(0.55–0.88)	(0.97–1.05)	(0.33–0.67)	(0.95–1.02)	(0.36–0.72)
18–30 years (*n* = 851)	0.95	0.64	0.93	0.65	1.08	0.48	1.04	0.50	0.96	0.29	0.92	0.31
	(0.90–1.01)	(0.42–0.98)	(0.88–0.99)	(0.43–0.99)	(1.03–1.13)	(0.28–0.84)	(0.99–1.09)	(0.29–0.86)	(0.88–1.04)	(0.12–0.69)	(0.85–0.99)	(0.13–0.72)
31–49 years (*n* = 1,335)	1.02	0.46	1.00	0.47	1.13	0.69	1.08	0.74	1.04	0.58	1.00	0.66
	(0.97–1.08)	(0.26–0.81)	(0.94–1.05)	(0.27–0.84)	(1.09–1.17)	(0.48–0.99)	(1.05–1.12)	(0.53–1.05)	(0.97–1.11)	(0.32–1.06)	(0.93–1.07)	(0.38–1.16)
≥50 years (*n* = 1,325)	0.99	0.95	0.98	0.96	1.14	0.78	1.12	0.81	1.02	0.57	1.00	0.62
	(0.95–1.04)	(0.75–1.21)	(0.94–1.03)	(0.75–1.22)	(1.10–1.18)	(0.57–1.06)	(1.08–1.15)	(0.61–1.09)	(0.96–1.07)	(0.34–0.96)	(0.94–1.05)	(0.38–1.01)
Men (*n* = 1,610)	1.00	0.68	0.99	0.70	1.10	0.62	1.08	0.65	1.01	0.44	0.99	0.48
	(0.96–1.04)	(0.49–0.94)	(0.94–1.03)	(0.51–0.97)	(1.07–1.13)	(0.43–0.88)	(1.05–1.11)	(0.46–0.92)	(0.96–1.06)	(0.25–0.76)	(0.94–1.04)	(0.28–0.83)
Women (*n* = 1,901)	0.98	0.77	0.97	0.78	1.14	0.72	1.10	0.76	1.02	0.51	0.98	0.55
	(0.94–1.02)	(0.58–1.02)	(0.93–1.01)	(0.59–1.03)	(1.10–1.18)	(0.52–0.98)	(1.06–1.13)	(0.56–1.03)	(0.97–1.09)	(0.31–0.84)	(0.92–1.03)	(0.34–0.88)
**NH-American Indian/Alaska Native, overall (** ***n*** ** = 172)**	0.94	0.47	0.95	0.47	0.93	0.51	0.93	0.55	1.01	0.88	1.01	0.98
	(0.84–1.04)	(0.21–1.06)	(0.85–1.06)	(0.21–1.05)	(0.84–1.03)	(0.17–1.51)	(0.84–1.02)	(0.20–1.49)	(0.88–1.17)	(0.44–1.76)	(0.88–1.16)	(0.45–2.14)
18–30 years (*n* = 35)	0.98	0.49	1.03	0.51	0.76	0.22	0.79	0.29	0.82	0.93	0.87	1.41
	(0.82–1.17)	(0.11–2.25)	(0.86–1.24)	(0.11–2.28)	(0.52–1.09)	(0.03–1.91)	(0.59–1.08)	(0.04–2.24)	(0.61–1.11)	(0.42–2.06)	(0.62–1.21)	(0.57–3.5)
31–49 years (*n* = 54)	NE	NE	NE	NE	NE	NE	NE	NE	NE	NE	NE	NE
≥50 years (*n* = 83)	0.94	0.54	0.93	0.54	1.03	1.03	1.01	0.98	1.06	0.81	1.03	0.75
	(0.78–1.13)	(0.17–1.71)	(0.78–1.12)	(0.17–1.70)	(0.87–1.22)	(0.58–1.83)	(0.86–1.17)	(0.57–1.69)	(0.90–1.24)	(0.37–1.74)	(0.86–1.22)	(0.36–1.58)
Men (*n* = 71)	0.96	0.46	0.98	0.48	0.98	0.15	0.98	0.17	1.09	0.77	1.09	0.96
	(0.79–1.17)	(0.15–1.39)	(0.81–1.19)	(0.16–1.40)	(0.82–1.17)	(0.02–1.20)	(0.84–1.15)	(0.02–1.31)	(0.88–1.35)	(0.24–2.48)	(0.89–1.33)	(0.28–3.30)
Women (*n* = 101)	0.92	0.52	0.94	0.50	0.89	1.46	0.88	1.33	0.96	1.02	0.96	0.96
	(0.8–1.07)	(0.29–0.92)	(0.81–1.08)	(0.28–0.89)	(0.77–1.02)	(1.26–1.69)	(0.77–0.99)	(1.18–1.51)	(0.77–1.20)	(0.68–1.52)	(0.79–1.17)	(0.63–1.47)
**NH-Asian, overall (** ***n*** ** = 1,531)**	0.98	0.96	0.94	0.94	1.16	1.02	1.09	1.02	1.09	0.78	1.00	0.79
	(0.95–1.02)	(0.72–1.28)	(0.91–0.98)	(0.72–1.24)	(1.13–1.18)	(0.77–1.34)	(1.06–1.12)	(0.78–1.33)	(1.04–1.14)	(0.48–1.28)	(0.96–1.05)	(0.48–1.28)
18–30 years (*n* = 272)	0.95	0.30	0.92	0.38	1.14	1.00	1.08	1.27	1.15	1.36	1.06	1.82
	(0.88–1.03)	(0.04–2.37)	(0.85–0.99)	(0.06–2.42)	(1.08–1.21)	(0.55–1.80)	(1.01–1.14)	(0.55–2.96)	(1.03–1.29)	(0.77–2.39)	(0.95–1.18)	(0.74–4.47)
31–49 years (*n* = 638)	1.01	1.04	0.98	1.00	1.13	0.98	1.06	0.94	1.13	0.80	1.04	0.77
	(0.96–1.08)	(0.66–1.63)	(0.92–1.04)	(0.64–1.57)	(1.10–1.17)	(0.52–1.83)	(1.03–1.10)	(0.51–1.74)	(1.05–1.22)	(0.27–2.41)	(0.96–1.12)	(0.28–2.12)
≥50 years (*n* = 621)	0.96	1.04	0.93	1.00	1.17	1.02	1.11	1.00	1.04	0.74	0.97	0.74
	(0.91–1.02)	(0.76–1.40)	(0.88–0.99)	(0.74–1.35)	(1.11–1.22)	(0.75–1.39)	(1.06–1.16)	(0.76–1.32)	(0.97–1.12)	(0.37–1.46)	(0.91–1.04)	(0.38–1.43)
Men (*n* = 669)	1.01	0.96	0.97	0.98	1.13	0.98	1.10	1.04	1.11	0.72	1.05	0.78
	(0.96–1.07)	(0.62–1.49)	(0.92–1.02)	(0.66–1.45)	(1.10–1.17)	(0.64–1.50)	(1.06–1.13)	(0.67–1.61)	(1.04–1.18)	(0.33–1.54)	(0.99–1.12)	(0.35–1.78)
Women (*n* = 862)	0.96	0.96	0.92	0.93	1.18	1.04	1.09	1.00	1.07	0.84	0.97	0.80
	(0.91–1.01)	(0.66–1.40)	(0.87–0.97)	(0.64–1.35)	(1.13–1.22)	(0.73–1.49)	(1.05–1.13)	(0.73–1.37)	(1.00–1.15.00.15)	(0.43–1.65)	(0.90–1.04)	(0.43–1.48)
**NH-Black/African American, overall (** ***n*** ** = 2,664)**	0.86	0.63	0.86	0.64	1.10	0.86	1.07	0.90	0.99	0.59	0.96	0.63
	(0.83–0.89)	(0.49–0.80)	(0.83–0.90)	(0.50–0.82)	(1.07–1.13)	(0.72–1.03)	(1.04–1.10)	(0.75–1.07)	(0.94–1.03)	(0.41–0.85)	(0.92–1.00)	(0.45–0.88)
18–30 years (*n* = 413)	0.87	0.73	0.86	0.74	1.07	1.07	1.01	1.08	0.92	0.69	0.87	0.70
	(0.80–0.94)	(0.48–1.13)	(0.79–0.93)	(0.49–1.12)	(1.00–1.13)	(0.84–1.36)	(0.95–1.08)	(0.84–1.40)	(0.82–1.04)	(0.35–1.37)	(0.77–0.97)	(0.38–1.26)
31–49 years (*n* = 792)	0.85	0.52	0.85	0.53	1.11	0.48	1.08	0.49	1.06	0.20	1.02	0.21
	(0.79–0.92)	(0.30–0.92)	(0.79–0.92)	(0.31–0.92)	(1.06–1.16)	(0.28–0.83)	(1.03–1.12)	(0.29–0.83)	(0.96–1.16)	(0.07–0.56)	(0.94–1.12)	(0.08–0.58)
≥50 years (*n* = 1,459)	0.88	0.63	0.88	0.65	1.13	0.97	1.10	1.03	0.99	0.76	0.97	0.84
	(0.84–0.93)	(0.45–0.89)	(0.83–0.93)	(0.46–0.91)	(1.09–1.16)	(0.78–1.22)	(1.06–1.14)	(0.82–1.29)	(0.94–1.05)	(0.52–1.11)	(0.92–1.02)	(0.58–1.21)
Men (*n* = 1,083)	0.86	0.57	0.85	0.58	1.07	0.84	1.05	0.87	1.00	0.52	0.98	0.55
	(0.81–0.91)	(0.40–0.80)	(0.80–0.90)	(0.41–0.81)	(1.03–1.11)	(0.67–1.05)	(1.02–1.09)	(0.70–1.08)	(0.94–1.07)	(0.30–0.90)	(0.92–1.04)	(0.33–0.91)
Women (*n* = 1,581)	0.86	0.70	0.87	0.71	1.13	0.90	1.09	0.94	0.98	0.69	0.95	0.74
	(0.82–0.91)	(0.49–0.98)	(0.82–0.92)	(0.50–1.00)	(1.09–1.18)	(0.68–1.19)	(1.05–1.14)	(0.71–1.25)	(0.92–1.05)	(0.43–1.10)	(0.89–1.01)	(0.47–1.16)
**NH-Multiracial or other** ^**e**^ **group, overall (*n* = 456)**	0.89	0.59	0.88	0.61	1.01	0.60	1.00	0.62	0.97	0.78	0.96	0.82
	(0.81–0.97)	(0.34–1.02)	(0.81–0.97)	(0.36–1.03)	(0.95–1.08)	(0.36–0.99)	(0.94–1.07)	(0.39–0.99)	(0.87–1.09)	(0.44–1.38)	(0.86–1.06)	(0.46–1.45)
18–30 years (*n* = 139)	0.99	0.21	0.97	0.22	NE	NE	NE	NE	1.00	0.22	0.97	0.30
	(0.87–1.11)	(0.05–0.88)	(0.86–1.10)	(0.05–0.97)					(0.83–1.20)	(0.03–1.51)	(0.82–1.15)	(0.04–2.21)
31–49 years (*n* = 160)	0.73	0.57	0.74	0.58	1.00	0.96	0.99	0.90	1.01	0.99	1.00	0.90
	(0.61–0.89)	(0.20–1.66)	(0.61–0.90)	(0.21–1.60)	(0.91–1.10)	(0.54–1.71)	(0.90–1.09)	(0.52–1.53)	(0.84–1.22)	(0.36–2.72)	(0.84–1.20)	(0.32–2.53)
≥50 years (*n* = 157)	0.87	0.98	0.87	0.98	0.99	0.66	0.98	0.68	0.86	1.05	0.84	1.12
	(0.74–1.02)	(0.63–1.53)	(0.74–1.01)	(0.63–1.52)	(0.86–1.13)	(0.32–1.36)	(0.86–1.12)	(0.33–1.39)	(0.72–1.03)	(0.59–1.85)	(0.71–1.01)	(0.61–2.06)
Men (*n* = 226)	0.85	0.76	0.85	0.81	1.00	0.51	1.00	0.53	0.92	0.72	0.92	0.77
	(0.74–0.97)	(0.43–1.35)	(0.74–0.97)	(0.48–1.38)	(0.92–1.08)	(0.22–1.19)	(0.92–1.08)	(0.24–1.19)	(0.79–1.08)	(0.33–1.58)	(0.79–1.07)	(0.36–1.62)
Women (*n* = 230)	0.93	0.34	0.93	0.33	1.04	0.73	1.01	0.75	1.04	0.86	1.01	0.90
	(0.83–1.05)	(0.12–0.95)	(0.83–1.04)	(0.12–0.93)	(0.94–1.16)	(0.38–1.41)	(0.92–1.12)	(0.41–1.38)	(0.89–1.22)	(0.41–1.85)	(0.87–1.17)	(0.38–2.13)

Abbreviations: NH (non-Hispanic), NE (not able to estimate).

^a^ Infrequent insomnia symptoms (yes) defined as difficulty falling or staying asleep never/some days in past 30 days.

^b^ Restorative sleep (yes) defined as most days/every day waking up feeling rested in the past 30 days.

^c^ Model 1 is adjusted for age (years) when not stratified by age, sex (man, woman), marital status (divorced/widowed, single/no live-in partner, married/living with partner/co-habituating), educational attainment (≤ high school, some college, ≥ college), employment status (employed - currently working, employed - not currently working, not employed - homemaker, not employed - going to school, not employed - retired, not employed - not able to work, not employed - looking for work, not employed - other), and general health status (fair/poor, good/very good/excellent). Model 2 is adjusted for covariates in Model 1 and BMI (underweight (< 18.5 kg/m), recommended (18.5–24.9 kg/m), overweight (25–29.9 kg/m), obesity (≥ 30 kg/m)), leisure-time physical activity (inactive, insufficiently active, sufficiently active), smoking status (never/quit > 12 months prior to interview, former, current), alcohol consumption (current, former, lifetime abstainer), and depression (yes, no). Models in the overall sample are additionally adjusted for race and ethnicity (Hispanic/Latino, NH-American Indian/Alaska Native, NH-Asian, NH-Black/African American, NH-Multiracial or other group, NH-White).

^d^ Life satisfaction was ascertained based on the questions, ‘In general, how satisfied are you with your life? Are you very satisfied, satisfied, dissatisfied, or very dissatisfied?‘. Life satisfaction was dichotomized as yes (a response of ‘satisfied’ or ‘very satisfied’) vs. no (a response of ‘very dissatisfied’ or ‘dissatisfied’).

^e^ NH-Other single and multiple races and ‘Other group’ is defined as persons identifying with racial groups not explicitly listed in the standard categories.

## Discussion

This study is the first, to our knowledge, to leverage a large, nationally-representative sample of U.S. adults to investigate associations between life satisfaction and favorable sleep health. Favorable sleep was defined as meeting sleep duration recommendations, reporting infrequent insomnia symptoms, and experiencing restorative sleep. Age, sex, and race along with ethnicity did not significantly modify overall associations. The relationship between life satisfaction and sleep outcomes remained consistent across demographic groups. However, modest variation in the magnitude of associations was observed. For instance, the association between life satisfaction and restorative sleep appeared slightly stronger among younger adults. Associations between life satisfaction and higher prevalence of recommended sleep, infrequent insomnia symptoms, and restorative sleep were comparable across sexes. Similarly, race and ethnicity did not significantly modify overall associations. However, lower prevalence of favorable sleep health outcomes were observed among Hispanic/Latino adults who reported satisfaction with life or dissatisfaction with life compared to NH-White adults satisfied with life. These patterns were consistent across age and sex. In comparison to NH-White counterparts who reported satisfaction with life, only Hispanic/Latino adults reporting dissatisfaction with life had lower prevalence of all favorable sleep health outcomes. While the prevalence of favorable sleep health outcomes did not vary across age and sex groups of NH-Black adults who reported satisfaction with life, all NH-Black adults satisfied with life had a lower prevalence of recommended sleep duration and restorative sleep compared to NH-White adults reporting satisfaction with life. However, differences were widest when NH-Black adults reported dissatisfaction with life. Additionally, associations among NH-Asian, NH-American Indian/Alaska Native, and NH-Multiracial and other adults did not vary by age and sex.

Plausible psychological mechanisms have been proposed in prior literature to explain the bidirectional relationship between life satisfaction and sleep. To our knowledge, no prior U.S.-based studies have examined the association between life satisfaction and sleep outcomes. Nevertheless, our findings are directionally consistent with evidence from cohorts in German, Swedish, Czech, South Korea, and China that have reported reciprocal associations between sleep quality and life satisfaction during key life transitions^10,11,14,15,17^. For instance, among young adults, several European cohorts have documented modest but significant associations in the predicted directions.

While the current study is the first nationally representative U.S. study investigating life satisfaction and sleep health, prior studies from China, Finland, and Iran have reported consistent associations between sleep and life satisfaction in the context of aging and retirement transitions^16,18–20^. Across multiple Chinese cohorts, there were significant positive correlations between sleep quality and life satisfaction, with longitudinal analyses demonstrating reciprocal associations between sleep duration or sleep quality and life satisfaction^18,20^. Specifically, prior studies reported modest standardized improvements in life satisfaction with decreasing sleep problems during retirement^16^, small reciprocal longitudinal effects between sleep duration or quality and life satisfaction in older adults^18,20^, elevated odds of life dissatisfaction among short or poor sleepers partially mediated by depression^19^, and modest associations were observed between sleep quality dimensions and life satisfaction^36^. In the current study, higher overall life satisfaction was associated with more favorable sleep outcomes. The magnitude of associations was modestly stronger for restorative sleep among young adults than older adults, a pattern that is directionally consistent with prior studies but suggests generally stronger and more direct relationships than previously reported. Shifts in priorities or lifestyle factors such as attending college or transitioning into retirement can alleviate stress and promote sleep health^16,18–20,36^.

Furthermore, sex did not modify associations. This pattern suggests that the association between life satisfaction and sleep health is broadly similar among men and women. This may indicate that the underlying psychosocial and physiologic pathways linking life satisfaction to sleep health may operate similarly for men and women. Nonetheless several biological and social factors including hormonal regulation, stress responses, and differential exposure to social and structural conditions^24,33,37,38^ have been proposed in prior literature as influences on these relationships. However, given the comparable associations observed among men and women in this study, these factors may not have exerted a strong influence on the associations between life satisfaction and recommended sleep duration, infrequent insomnia symptoms, or restorative sleep.

Differences in income, housing stability, and neighborhood environments by race and ethnicity are well documented drivers of variability in sleep health^39,40^. Our findings suggest that these structural disadvantages may be partially attenuated among individuals who report high life satisfaction. In this study, Hispanic/Latino and NH-Black adults dissatisfied with life had lower prevalence of recommended sleep duration and restorative sleep and comparable prevalence of infrequent insomnia symptoms compared to NH-White adults satisfied with life. These racial and ethnic differences are consistent with prior studies that have documented directionally consistent associations between life satisfaction and sleep health across population groups^41^. Prior literature has suggested that familial and social support systems may be associated with reduced stress-related sleep disruptions^41–44^. Conversely, despite reporting life satisfaction, NH-Asian, NH-Black, and NH-multiracial/Other adults had lower prevalence of recommended sleep relative to NH-White adults, which may suggest socioeconomic and social stressors that may contribute to poorer sleep health^25,26^. However, life satisfaction alone may not fully offset the influence of persistent sociocultural and environmental stressors, underscoring the importance of considering both psychological and contextual determinants. For instance, bottom-up theories which conceptualize life satisfaction as the aggregation of satisfaction across multiple domains such as health, finances, and relationships. In contrast, top-down theories emphasize the role of stable personality traits and cognitive appraisals^45–49^. Further investigation is warranted to elucidate the contextual and psychosocial mechanisms underlying these divergent patterns.

This study has several limitations. First, the cross-sectional design precludes causal inference and limits our ability to determine the temporality between life satisfaction and sleep outcomes. Longitudinal studies can help establish directionality. Second, reliance on self-reported sleep introduces potential recall and social desirability bias, which may lead to nondifferential misclassification. Such misclassification would likely bias estimates toward the null by attenuating true associations between life satisfaction and sleep outcomes^50^. Future studies using objective measures (e.g., actigraphy or polysomnography) could improve validity. Third, unmeasured or residual confounding may persist despite adjustment for key covariates; relevant lifestyle (e.g., religiosity, resiliency and social support) or psychosocial factors (e.g., trauma exposure) may not have been captured. Residual confounding may lead to overestimation or underestimation of the true relationships. Fourth, income was not assessed as a mediator in the model adjustment, although related variables (i.e., employment status, educational attainment) were included. While mediators are not typically adjusted for when estimating total effects, omitting income limits our ability to assess socioeconomic pathways through which life satisfaction may influence sleep. This may result in residual socioeconomic confounding and may limit interpretation of the mechanisms underlying the observed associations. Finally, data collection during the COVID-19 pandemic may have influenced both sleep and patterns of life satisfaction non-differentially. Pandemic-related stress, disruptions to daily routines, and changes in work or caregiving responsibilities may have been atypical, limiting generalizability to non-pandemic periods. These factors could bias associations toward the null by reducing variability in both life satisfaction and sleep outcomes, making true relationships more difficult to detect. Furthermore, although the NHIS is nationally representative of non-institutionalized U.S. adults, the results may not be generalizable to institutionalized populations, individuals experiencing homelessness, or individuals with severe mental illness or sleep disorders; Targeted studies in these populations are warranted to more fully characterize the relationship between life satisfaction and sleep health.

Despite the limitations, this study has notable strengths. We used a nationally-representative sample of U.S. adults to determine the associations between life satisfaction and sleep health across sociodemographic groups, strengthening external validity to individuals living in the U.S. The large sample size enables robust statistical analyses and supports examination of interrelated associations in the life satisfaction-sleep relationship, including assessment of effect modification by key sociodemographic factors and of potential mediators of the life satisfaction and sleep relationship.

In conclusion, life satisfaction was associated with recommended sleep duration, infrequent insomnia symptoms, and restorative sleep in a large, nationally representative sample of U.S. adults. These associations were largely consistent across age, sex, and race along with ethnicity, though magnitudes varied. Public health strategies that enhance life satisfaction—through psychological support, socioeconomic security, and cultural resilience—may be relevant for future research on sleep health promotion. Future longitudinal studies incorporating objective sleep metrics and stress biomarkers are warranted to clarify temporal relationships and inform targeted public health promotion strategies.

## Supplementary Information

Below is the link to the electronic supplementary material.

Supplementary Material 1

## Data Availability

Participant data that underlie the primary results reported in this article are publicly available. All data release requests require a proposal including a data dictionary. For additional details on data access refer to the [National Health Interview Survey.](https:/nhis.ipums.org/nhis/userNotes_NHISinFSRDC.shtml).
