# Supplementary material for: Satisfaction with life in relation to sleep health among a nationally representative sample of U.S. adults
Source: Sci Rep. 2026 May 15;16:15189. doi: 10.1038/s41598-026-41318-4 (PMC13179357; doi:10.1038/s41598-026-41318-4)
Supplement: Supplementary file 1 — Supplementary Material 1 [file 41598_2026_41318_MOESM1_ESM.docx]

**Supplemental Figure 1. Flow chart of study population selection**

NHIS 2022

sample of adults

**(N=27,651)**

Final analytic sample adults

**N=25,090**

- Missing data
  - All sleep measures (n = 1,044)
  - Life satisfaction (n = 58)
  - Sex (n =3)
  - Race and ethnicity (n=0)
  - Age (n=56)
  - Potential confounders (n= 1,400)
- **Total excluded (n =2,561)**

**Excluded**

**Supplemental Table 1. Comparison of included and excluded study participants, National Health Interview Survey, 2022, (N=27,651)**

|  | Included n=25,090 (90.7%) | Excluded  n=2,561 (9.3%) | Chi-square or t-test p-value |
| --- | --- | --- | --- |
| Sociodemographic Characteristics |  |  |  |
| Age (years), mean (SE) | 48.1 (.17) | 66.6 (2.4) | <0.001 |
| Age group |  |  | 0.552 |
| 18-30 | 21.8 | 20.8 |  |
| 31-49 | 31.5 | 32.5 |  |
| ≥ 50 | 46.7 | 46.7 |  |
| Sex |  |  | <0.001 |
| Men | 49.1 | 44.5 |  |
| Women | 50.9 | 55.5 |  |
| Race/ethnicity |  |  | <0.001 |
| Hispanic/Latino | 16.9 | 20.4 |  |
| NH-American Indian/Alaska Native | 0.8 | 0.7 |  |
| NH-Asian | 6.1 | 5.3 |  |
| NH-Black/African American | 11.2 | 18.4 |  |
| NH-Multiracial or other group ^a^ | 2.0 | 2.0 |  |
| NH-White | 62.9 | 53.2 |  |
| Educational Attainment |  |  | <0.001 |
| ≤High School | 37.2 | 46.3 |  |
| Some College | 29.6 | 27.9 |  |
| ≥College | 33.2 | 25.8 |  |
| Employment status |  |  | <0.001 |
| Employed, currently working | 63.7 | 59.1 |  |
| Employed, not currently working | 0.6 | 0.5 |  |
| Not employed, Homemaker | 4.7 | 5.3 |  |
| Not employed, Going to school | 2.3 | 3.5 |  |
| Not employed, Retired | 18.9 | 18.9 |  |
| Not employed, not able to work | 6.0 | 9.4 |  |
| Not employed, looking for work | 2.2 | 2.1 |  |
| Not employed, Other | 1.6 | 1.2 |  |
| Marital status |  |  | <0.001 |
| Divorced/widowed | 17.2 | 19.3 |  |
| Single/no live-in partner | 23.6 | 28.8 |  |
| Married/living with partner/co-habiting | 59.2 | 51.9 |  |
| Region of residence |  |  | <0.001 |
| Northeast | 17.2 | 21.2 |  |
| Midwest | 21.0 | 17.4 |  |
| South | 37.9 | 39.7 |  |
| West | 23.8 | 21.6 |  |
| Health Behaviors |  |  |  |
| Smoking status |  |  | 0.161 |
| Never/quit >12 months prior | 87.3 | 85.7 |  |
| Former/quit≤12 months ago | 1.2 | 1.1 |  |
| Current | 11.5 | 13.2 |  |
| Alcohol consumption |  |  | <0.001 |
| Current (≥1 drink past year) | 69.9 | 61.3 |  |
| Former (no drinks past year) | 17.0 | 18.5 |  |
| Lifetime abstinence (<12 drinks in life) | 13.0 | 20.2 |  |
| Leisure-time physical activity ^b^ |  |  | <0.001 |
| Inactive | 26.5 | 38.0 |  |
| Insufficiently active | 25.6 | 24.1 |  |
| Sufficiently active | 47.8 | 37.9 |  |
| Usual sleep duration |  |  | 0.045 |
| Short (<7 hours) | 30.3 | 27.2 |  |
| Recommended (≥7 hours) | 69.7 | 72.8 |  |
| Infrequent Insomnia symptoms (yes) ^c^ | 23.3 | 22.7 | 0.691 |
| Restorative sleep (yes) ^d^ | 43.0 | 45.1 | 0.163 |
| Clinical Characteristics |  |  |  |
| Ever had depression (yes) ^e^ | 18.5 | 16.4 | 0.028 |
| Body mass index category |  |  | 0.391 |
| Underweight (<18.5 kg/m^2^) | 1.7 | 1.8 |  |
| Recommended (18.5-<25 kg/m^2^) | 31.2 | 32.1 |  |
| Overweight (25-<30 kg/m^2^) | 33.7 | 35.0 |  |
| Obesity (≥30 kg/m^2^) | 33.4 | 31.1 |  |
| General health status |  |  | <0.001 |
| Fair/poor | 13.9 | 19.8 |  |
| Good/very good/excellent | 86.1 | 80.2 |  |
| Abbreviations: SE (standard error), NH (non-Hispanic) | | | |
| Note: Data are presented as column percentages or means and standard errors. Percentages may not sum to 100 due to missing rounding. All estimates are weighted for the survey's complex sampling design. All estimates are age-standardized to the U.S. 2020 population, except for age. | | | |
| ^a^ NH-Other single and multiple races and ‘Other group’ is defined as persons identifying with racial groups not explicitly listed in the standard categories.  ^b^ Leisure-time physical activity was defined using the 2018 Health and Human Services Physical Activity guidelines which state “recommend that adults complete at least 150 minutes to 300 minutes of moderate-intensity activity, or 75 minutes to 150 minutes of vigorous-intensity aerobic activity per week, as well as moderate or greater intensity muscle strengthening activities on two or more days a week."  ^c^ Infrequent Insomnia symptoms (yes) defined as difficulty falling or staying asleep never/some days. | | | |
| ^d^ Restorative sleep (yes) defined as most days/every day waking up feeling rested in the past 30 days. | | | |
| ^e^ Depression was defined by the 2019 Field Representative's Manual as a major depressive disorder or as clinical depression that is a common but serious mood disorder. It causes severe symptoms that affect how you feel, think, and handle daily activities, such as sleeping, eating, or working.” | | | |

**Supplemental Table 2. Variable name and descriptions, National Health Interview Survey, 2022**

| **Variable Name** | **Variable Description** |
| --- | --- |
| age | age |
| alcstat1 | alcohol drinking status |
| bmicat | categorical body mass index |
| depressev | ever had depression |
| educ | educational attainment |
| empstat | employment status in past 1 to 2 weeks |
| health | health status |
| hisprace | Hispanic ethnicity and self-reported race |
| hrsleep | usual hours sleep per day |
| marstcohab | marital status, including living with partner |
| pa18aer | physical activity met 2018 guidelines for aerobic activity |
| quityrs | time (years) since quit smoking |
| region | region of residence |
| sampweight | sample person weight |
| satisfied | how satisfied with life |
| sex | sex |
| sleepfallfrq | how often had trouble falling asleep, past 30 days |
| sleeprestfrq | how often woke up feeling well rested, past 30 days |
| sleepstayfrq | how often had trouble staying asleep, past 30 days |
| smokestatus2 | cigarette smoking status |
| whynowk2 | main reason not working last week |

Lynn A. Blewett, Julia A. Rivera Drew, Andrew Fenelon, Miriam L. King, Kari C.W. Williams, Daniel Backman, Etienne Breton, Grace Cooper, and Stephanie Richards. IPUMS Health Surveys: National Health Interview Survey, Version 8.1 [dataset]. Minneapolis, MN: IPUMS, 2025. https://doi.org/10.18128/D071.V8.

For the 2022 NHIS Survey Description, please visit: <https://ftp.cdc.gov/pub/Health_Statistics/NCHS/Dataset_Documentation/NHIS/2022/srvydesc-508.pdf>.

**Supplemental Table 3 Study population characteristics, overall and by age, National Health Interview Survey, 2022, (N=25,090)**

|  | Total  n=25,090 (100%) | | | 18-30 years  n=3,639 (14.5%) | | | 31-49 years  n=7,198 (28.7%) | | | ≥ 50 years  n=14,253 (56.8%) | | |
| --- | --- | --- | --- | --- | --- | --- | --- | --- | --- | --- | --- | --- |
|  |  | Life satisfaction ^a^ | |  | Life satisfaction ^a^ | |  | Life satisfaction ^a^ | |  | Life satisfaction ^a^ | |
|  | All  n=25,090 (100%) | Yes n=23,997 (95.6%) | No  n=1,093 (4.4%) | All  n=3,639 (100%) | Yes  n=3,515 (96.6%) | No  n=124 (3.4%) | All  n=7,198 (100%) | Yes  n=6,941 (96.4%) | No  n=257 (3.6%) | All  n=14,253 (100%) | Yes n=13,541 (95.0%) | No  n=712 (5.0%) |
| Sociodemographic Characteristics |  | | |  | | |  | | |  | | |
| Age (years), mean (SE) | 48.1(.17) | 48.0(.17) | 50.5(.75) | 24.1(.07) | 24.1(.07) | 24.1(.37) | 39.7(.07) | 39.7(.08) | 39.9(.39) | 64.9(.10) | 64.9(.11) | 65.4(.47) |
| Race and ethnicity |  | | |  | | |  | | |  | | |
| Hispanic/Latino | 16.9 | 17.1 | 13.6 | 22.6 | 22.7 | 19.7 | 20.0 | 20.2 | 13.7 | 12.2 | 12.3 | 10.5 |
| NH-American Indian/Alaska Native | 0.8 | 0.8 | 1.0 | 1.2 | 1.2 | 2.4 | 0.8 | 0.8 | 0.2 | 0.6 | 0.6 | 0.7 |
| NH-Asian | 6.1 | 6.3 | 2.7 | 5.7 | 5.9 | 1.0 | 7.7 | 7.8 | 3.5 | 5.3 | 5.4 | 3.1 |
| NH-Black/African American | 11.2 | 11.1 | 13.5 | 12.5 | 12.2 | 19.2 | 12.0 | 11.9 | 14.0 | 10.0 | 10.0 | 10.4 |
| NH-Multiracial or other group ^b^ | 2.0 | 2.0 | 3.4 | 3.7 | 3.7 | 4.3 | 2.1 | 2.0 | 5.0 | 1.2 | 1.1 | 1.9 |
| NH-White | 62.9 | 62.8 | 65.9 | 54.3 | 54.4 | 53.3 | 57.5 | 57.2 | 63.6 | 70.8 | 70.6 | 73.5 |
| Sex |  | | |  | | |  | | |  | | |
| Men | 49.1 | 49.0 | 51.3 | 50.6 | 50.2 | 63.2 | 49.8 | 49.8 | 49.9 | 47.9 | 48.0 | 46.2 |
| Women | 50.9 | 51.0 | 48.7 | 49.4 | 49.8 | 36.8 | 50.2 | 50.2 | 50.1 | 52.1 | 52.0 | 53.8 |
| Educational Attainment |  | | |  | | |  | | |  | | |
| ≤ High School | 37.2 | 36.8 | 47.3 | 40.9 | 40.6 | 50.2 | 31.2 | 30.7 | 45.0 | 39.5 | 39.2 | 47.2 |
| Some college | 29.6 | 29.5 | 33.6 | 33.7 | 33.6 | 36.2 | 28.2 | 28.0 | 34.1 | 28.7 | 28.5 | 31.8 |
| ≥ College | 33.2 | 33.8 | 19.2 | 25.4 | 25.8 | 13.7 | 40.6 | 41.3 | 20.9 | 31.8 | 32.3 | 21.0 |
| Employment status |  | | |  | | |  | | |  | | |
| Employed, currently working | 63.7 | 64.6 | 41.0 | 75.5 | 76.0 | 62.4 | 82.1 | 83.1 | 52.7 | 45.2 | 46.3 | 23.1 |
| Employed, not currently working | 0.6 | 0.6 | 0.9 | 0.5 | 0.5 | 0.8 | 0.7 | 0.6 | 1.1 | 0.6 | 0.6 | 0.7 |
| Not employed, Homemaker | 4.7 | 4.7 | 4.7 | 5.1 | 5.0 | 7.5 | 7.8 | 7.8 | 7.1 | 2.5 | 2.5 | 1.8 |
| Not employed, Going to school | 2.3 | 2.4 | 1.6 | 9.8 | 9.9 | 7.0 | 0.5 | 0.5 | 0.0 | 0.1 | 0.1 | 0.0 |
| Not employed, Retired | 18.9 | 19.0 | 16.8 | 0.0 | 0.0 | 0.0 | 0.3 | 0.3 | 0.0 | 40.9 | 41.1 | 36.3 |
| Not employed, not able to work | 6.0 | 5.1 | 25.0 | 2.4 | 2.4 | 3.5 | 4.7 | 3.9 | 28.1 | 8.4 | 7.2 | 33.0 |
| Not employed, looking for work | 2.2 | 2.0 | 6.3 | 4.5 | 4.1 | 12.6 | 2.1 | 1.9 | 5.4 | 1.2 | 1.1 | 3.7 |
| Not employed, Other | 1.6 | 1.5 | 3.8 | 2.3 | 2.1 | 6.2 | 1.9 | 1.7 | 5.6 | 1.1 | 1.1 | 1.4 |
| Marital status |  | | |  | | |  | | |  | | |
| Divorced/widowed | 17.2 | 16.7 | 28.0 | 1.9 | 1.8 | 4.2 | 11.0 | 10.7 | 21.5 | 28.7 | 27.9 | 43.9 |
| Single/no live-in partner | 23.6 | 23.1 | 37.4 | 65.9 | 65.4 | 77.7 | 17.9 | 17.1 | 41.7 | 7.7 | 7.3 | 14.7 |
| Married/living with partner/co-habited | 59.2 | 60.2 | 34.6 | 32.2 | 32.7 | 18.1 | 71.1 | 72.2 | 36.8 | 63.7 | 64.8 | 41.4 |
| Region of residence |  | | |  | | |  | | |  | | |
| Northeast | 17.2 | 17.2 | 17.8 | 17.6 | 17.4 | 23.0 | 16.1 | 16.1 | 14.0 | 17.8 | 17.8 | 17.6 |
| Midwest | 21.0 | 21.0 | 22.1 | 21.1 | 21.2 | 20.9 | 20.2 | 20.1 | 23.0 | 21.5 | 21.5 | 22.0 |
| South | 37.9 | 38.0 | 35.4 | 37.9 | 38.2 | 29.4 | 37.9 | 37.9 | 38.5 | 37.9 | 38.0 | 36.8 |
| West | 23.8 | 23.8 | 24.7 | 23.4 | 23.3 | 26.8 | 25.8 | 25.9 | 24.6 | 22.7 | 22.7 | 23.6 |
| **Health Behaviors** |  | | |  | | |  | | |  | | |
| Smoking status |  | | |  | | |  | | |  | | |
| Never/quit>12 months prior | 87.3 | 87.8 | 73.9 | 91.0 | 91.4 | 81.9 | 84.8 | 85.7 | 59.7 | 87.2 | 87.6 | 78.9 |
| Former/quit≤12 months ago | 1.2 | 1.2 | 2.4 | 1.8 | 1.7 | 3.4 | 1.4 | 1.4 | 1.0 | 0.8 | 0.7 | 2.8 |
| Current | 11.5 | 11.1 | 23.7 | 7.2 | 6.9 | 14.8 | 13.9 | 13.0 | 39.2 | 12.0 | 11.6 | 18.3 |
| Alcohol consumption |  | | |  | | |  | | |  | | |
| Current (≥1 drink past year) | 69.9 | 70.2 | 64.4 | 72.4 | 72.2 | 77.5 | 76.9 | 77.1 | 71.9 | 64.0 | 64.5 | 53.2 |
| Former (no drinks past year) | 17.0 | 16.7 | 23.8 | 8.3 | 8.2 | 9.2 | 12.2 | 12.0 | 17.6 | 24.5 | 23.9 | 35.1 |
| Lifetime abstinence (<12 drinks in life) | 13.0 | 13.1 | 11.7 | 19.3 | 19.6 | 13.3 | 10.9 | 10.9 | 10.5 | 11.6 | 11.6 | 11.7 |
| Leisure-time physical activity ^c^ |  | | |  | | |  | | |  | | |
| Inactive | 26.5 | 25.8 | 44.3 | 19.0 | 18.9 | 20.9 | 22.7 | 22.0 | 43.5 | 32.7 | 31.6 | 56.4 |
| Insufficiently active | 25.6 | 25.7 | 23.4 | 22.9 | 22.9 | 25.5 | 27.6 | 27.8 | 22.5 | 25.5 | 25.6 | 22.9 |
| Sufficiently active | 47.8 | 48.5 | 32.3 | 58.1 | 58.2 | 53.6 | 49.6 | 50.2 | 34.0 | 41.8 | 42.8 | 20.8 |
| Usual sleep duration |  | | |  | | |  | | |  | | |
| Short (<7 hours) | 30.3 | 29.7 | 45.4 | 26.7 | 26.0 | 45.7 | 34.3 | 33.8 | 50.0 | 29.1 | 28.5 | 41.8 |
| Recommended (≥7 hours) | 69.7 | 70.3 | 54.6 | 73.3 | 74.0 | 54.3 | 65.7 | 66.2 | 50.0 | 70.9 | 71.5 | 58.2 |
| Infrequent insomnia symptoms (yes) ^d^ | 76.7 | 77.8 | 49.6 | 77.8 | 78.8 | 52.6 | 78.8 | 79.8 | 48.1 | 74.8 | 76.1 | 49.6 |
| Restorative sleep (yes) ^e^ | 57.0 | 58.3 | 25.7 | 54.6 | 55.9 | 21.6 | 51.9 | 53.0 | 21.8 | 61.6 | 63.1 | 30.5 |
| **Clinical Characteristics** |  | | |  | | |  | | |  | | |
| Ever had depression (yes) ^f^ | 18.5 | 16.9 | 55.2 | 21.0 | 19.7 | 54.0 | 18.0 | 16.7 | 56.1 | 17.5 | 15.7 | 55.2 |
| Body mass index category |  | | |  | | |  | | |  | | |
| Underweight (<18.5 kg/m^2^) | 1.7 | 1.6 | 2.8 | 3.5 | 3.5 | 3.5 | 0.9 | 0.9 | 2.3 | 1.3 | 1.2 | 2.8 |
| Recommended (18.5-<25 kg/m^2^) | 31.2 | 31.3 | 28.2 | 42.4 | 42.5 | 38.9 | 28.0 | 28.2 | 24.2 | 28.2 | 28.3 | 25.4 |
| Overweight (25-<30 kg/m^2^) | 33.7 | 34.0 | 27.0 | 28.3 | 28.5 | 24.4 | 34.0 | 34.4 | 20.7 | 36.1 | 36.3 | 32.0 |
| Obesity (≥30 kg/m^2^) | 33.4 | 33.0 | 42.0 | 25.7 | 25.4 | 33.1 | 37.1 | 36.5 | 52.9 | 34.4 | 34.1 | 39.8 |
| General health status |  | | |  | | |  | | |  | | |
| Fair/poor | 13.9 | 12.3 | 51.8 | 6.2 | 5.2 | 32.1 | 9.8 | 8.5 | 48.7 | 20.4 | 18.3 | 63.4 |
| Good/very good/excellent | 86.1 | 87.7 | 48.2 | 93.8 | 94.8 | 67.9 | 90.2 | 91.5 | 51.3 | 79.6 | 81.7 | 36.6 |
| Abbreviations: SE (standard error), NH (non-Hispanic) | | | | | | | | | | | | |
| Note: Data are presented as column percentages or means and standard errors. Percentages may not sum to 100 due to missing rounding. All estimates are weighted for the survey's complex sampling design. All estimates are age-standardized to theU.S.2020 population, except for age. | | | | | | | | | | | | |
| ^a^ Life satisfaction was ascertained based on the questions, 'In general, how satisfied are you with your life? Are you very satisfied, satisfied, dissatisfied, or very dissatisfied?'. Life satisfaction was dichotomized as yes (a response of 'satisfied' or 'very satisfied') vs. no (a response of 'very dissatisfied' or 'dissatisfied'). | | | | | | | | | | | | |
| ^b^ NH-Other single and multiple races and ‘Other group’ is defined as persons identifying with racial groups not explicitly listed in the standard categories.  ^c^ Leisure-time physical activity was defined using the 2018 Health and Human Services Physical Activity guidelines which state “recommend that adults complete at least 150 minutes to 300 minutes of moderate-intensity activity, or 75 minutes to 150 minutes of vigorous-intensity aerobic activity per week, as well as moderate or greater intensity muscle strengthening activities on two or more days a week."  ^d^ Infrequent Insomnia symptoms (yes) defined as difficulty falling or staying asleep never/some days. | | | | | | | | | | | | |
| ^e^ Restorative sleep (yes) defined as most days/every day waking up feeling rested in the past 30 days. | | | | | | | | | | | | |
| ^f^ Depression was defined by the 2019 Field Representative's Manual as a major depressive disorder or as clinical depression that is a common but serious mood disorder. It causes severe symptoms that affect how you feel, think, and handle daily activities, such as sleeping, eating, or working.” | | | | | | | | | | | | |

**Supplemental Table 4. Study population characteristics, overall and by race and ethnicity, National Health Interview Survey, 2022, (N=25,090)**

|  | Total  n=25,090 (100%) | | | Hispanic/Latino n=3,511 (14.0%) | | | NH-American Indian/Alaska Native n=172 (0.7%) | | | NH-Asian  n=1,531 (6.1%) | | | NH-Black/African American  n=2,664 (10.6%) | | | NH-Multiracial or other group ^a^  n=456 (1.8%) | | | NH-White  n=16,756 (66.8%) | | |
| --- | --- | --- | --- | --- | --- | --- | --- | --- | --- | --- | --- | --- | --- | --- | --- | --- | --- | --- | --- | --- | --- |
|  |  | Life satisfaction ^b^ | |  | Life satisfaction ^b^ | |  | Life satisfaction ^b^ | |  | Life satisfaction ^b^ | |  | Life satisfaction ^b^ | |  | Life satisfaction ^b^ | |  | Life satisfaction ^b^ | |
|  | All n=25,090 (100%) | Yes n=23,997 (95.6%) | No n=1,093 (4.4%) | All n=3,511 (100%) | Yes n=3,389 (96.5%) | No n=122 (3.5%) | All n=172 (100%) | Yes n=160 (93.0%) | No  n=12 (7.0%) | All n=1,531 (100%) | Yes n=1,500 (98.0%) | No  n=31 (2.0%) | All n=2,664 (100%) | Yes n=2,540 (95.3%) | No n=124 (4.7%) | All n=456 (100%) | Yes n=428 (93.9%) | No  n=28 (6.1%) | All n=16,756 (100%) | Yes n=15,980 (95.4%) | No n=776 (4.6%) |
| Sociodemographic Characteristics |  | | |  | | |  | | |  | | |  | | |  | | |  | | |
| Age (years), mean (SE) | 48.1(.17) | 48.0(.17) | 50.5(.75) | 42.6(.33) | 42.5(.33) | 46.4(2.3) | 43.5(1.5) | 43.7(1.5) | 39.0(6.1) | 46.3(.56) | 46.2(.57) | 53.0(2.7) | 46.0(.43) | 46.0(.44) | 44.7(2.0) | 39.0(.93) | 38.8(.96) | 41.9(3.1) | 50.4(.21) | 50.3(.21) | 52.9(.90) |
| 18-30 years | 21.8 | 21.9 | 20.0 | 29.1 | 29.1 | 29.9 | 33.7 | 32.8 | 51.8 | 20.3 | 20.5 | 7.5 | 24.4 | 24.1 | 29.4 | 40.2 | 41.1 | 26.9 | 18.8 | 18.9 | 16.0 |
| 31-49 years | 31.5 | 31.7 | 26.1 | 37.2 | 37.5 | 27.2 | 32.5 | 33.8 | 6.8 | 39.4 | 39.5 | 32.7 | 33.6 | 33.9 | 27.9 | 32.9 | 32.4 | 40.5 | 28.7 | 28.9 | 24.8 |
| ≥ 50 years | 46.7 | 46.4 | 53.9 | 33.7 | 33.4 | 42.9 | 33.8 | 33.4 | 41.4 | 40.3 | 40.0 | 59.8 | 42.0 | 42.0 | 42.6 | 26.9 | 26.5 | 32.5 | 52.5 | 52.2 | 59.2 |
| Sex |  | | |  | | |  | | |  | | |  | | |  | | |  | | |
| Men | 49.1 | 49.0 | 51.3 | 49.0 | 48.9 | 49.6 | 38.9 | 37.5 | 69.2 | 45.6 | 45.6 | 53.2 | 44.4 | 44.1 | 50.8 | 52.4 | 51.7 | 61.9 | 50.5 | 50.5 | 50.9 |
| Women | 50.9 | 51.0 | 48.7 | 51.0 | 51.1 | 50.4 | 61.1 | 62.5 | 30.8 | 54.4 | 54.4 | 46.8 | 55.6 | 55.9 | 49.2 | 47.6 | 48.3 | 38.1 | 49.5 | 49.5 | 49.1 |
| Educational Attainment |  | | |  | | |  | | |  | | |  | | |  | | |  | | |
| ≤ High School | 37.2 | 36.8 | 47.3 | 57.1 | 56.8 | 65.1 | 49.1 | 49.5 | 41.5 | 29.9 | 29.1 | 70.3 | 45.5 | 45.6 | 42.3 | 36.2 | 35.2 | 47.2 | 31.4 | 30.8 | 44.4 |
| Some college | 29.6 | 29.5 | 33.6 | 26.7 | 26.7 | 24.0 | 36.3 | 36.3 | 34.5 | 17.4 | 17.6 | 12.7 | 31.7 | 31.1 | 43.0 | 38.0 | 38.4 | 33.3 | 30.5 | 30.3 | 33.6 |
| ≥ College | 33.2 | 33.8 | 19.2 | 16.3 | 16.5 | 10.9 | 14.6 | 14.1 | 24.0 | 52.6 | 53.3 | 17.0 | 22.9 | 23.3 | 14.7 | 25.9 | 26.5 | 19.5 | 38.1 | 38.8 | 22.0 |
| Employment status |  | | |  | | |  | | |  | | |  | | |  | | |  | | |
| Employed, currently working | 63.7 | 64.6 | 41.0 | 64.4 | 64.9 | 45.4 | 49.5 | 51.1 | 13.9 | 62.5 | 63.1 | 31.9 | 61.2 | 62.2 | 40.7 | 62.8 | 65.0 | 31.9 | 64.6 | 65.7 | 41.0 |
| Employed, not currently working | 0.6 | 0.6 | 0.9 | 0.5 | 0.5 | 1.1 | 2.4 | 2.5 | 0.0 | 0.6 | 0.6 | 0.0 | 0.6 | 0.6 | 1.5 | 0.5 | 0.2 | 5.9 | 0.6 | 0.6 | 0.3 |
| Not employed, Homemaker | 4.7 | 4.7 | 4.7 | 7.1 | 7.2 | 2.5 | 8.7 | 9.1 | 0.0 | 6.9 | 6.8 | 8.4 | 2.7 | 2.6 | 5.5 | 4.9 | 5.2 | 0.0 | 4.2 | 4.2 | 5.0 |
| Not employed, Going to school | 2.3 | 2.4 | 1.6 | 2.0 | 2.1 | 0.0 | 2.2 | 2.3 | 0.0 | 5.1 | 5.1 | 0.0 | 2.6 | 2.5 | 3.3 | 2.6 | 2.7 | 2.2 | 2.1 | 2.1 | 1.7 |
| Not employed, Retired | 18.9 | 19.0 | 16.8 | 15.5 | 15.6 | 14.0 | 18.8 | 19.1 | 11.1 | 17.1 | 17.1 | 19.3 | 18.3 | 18.6 | 12.0 | 17.1 | 17.0 | 19.5 | 19.7 | 19.8 | 17.8 |
| Not employed, not able to work | 6.0 | 5.1 | 25.0 | 5.7 | 4.8 | 30.8 | 11.4 | 10.2 | 37.6 | 2.9 | 2.4 | 26.7 | 9.2 | 8.5 | 23.1 | 9.9 | 7.9 | 37.2 | 5.8 | 4.9 | 24.4 |
| Not employed, looking for work | 2.2 | 2.0 | 6.3 | 2.9 | 2.8 | 5.2 | 4.5 | 3.1 | 31.9 | 2.9 | 2.8 | 5.3 | 3.5 | 3.5 | 5.2 | 0.8 | 0.5 | 3.4 | 1.7 | 1.5 | 6.4 |
| Not employed, Other | 1.6 | 1.5 | 3.8 | 2.0 | 2.0 | 1.1 | 2.7 | 2.5 | 5.5 | 2.1 | 2.0 | 8.4 | 1.9 | 1.5 | 8.8 | 1.4 | 1.5 | 0.0 | 1.4 | 3.5 | 5.2 |
| Marital status |  | | |  | | |  | | |  | | |  | | |  | | |  | | |
| Divorced/widowed | 17.2 | 16.7 | 28.0 | 18.9 | 18.8 | 23.6 | 24.7 | 24.9 | 19.1 | 12.0 | 11.7 | 23.6 | 23.4 | 23.2 | 27.7 | 22.4 | 22.5 | 22.1 | 16.1 | 15.5 | 29.5 |
| Single/no live-in partner | 23.6 | 23.1 | 37.4 | 23.6 | 23.1 | 38.2 | 29.5 | 27.9 | 62.4 | 21.0 | 20.7 | 35.3 | 37.0 | 36.6 | 45.9 | 28.6 | 27.9 | 37.2 | 20.9 | 20.3 | 34.8 |
| Married/living with partner/co-habited | 59.2 | 60.2 | 34.6 | 57.5 | 58.1 | 38.3 | 45.8 | 47.2 | 18.6 | 67.0 | 67.6 | 41.1 | 39.6 | 40.2 | 26.4 | 49.0 | 49.6 | 40.7 | 63.0 | 64.2 | 35.7 |
| Region of residence |  | | |  | | |  | | |  | | |  | | |  | | |  | | |
| Northeast | 17.2 | 17.2 | 17.8 | 13.7 | 13.6 | 17.6 | 5.6 | 5.5 | 5.5 | 21.2 | 21.3 | 12.3 | 14.8 | 14.5 | 21.0 | 8.8 | 8.8 | 8.5 | 18.8 | 18.8 | 18.3 |
| Midwest | 21.0 | 21.0 | 22.1 | 8.1 | 8.1 | 9.3 | 14.3 | 14.3 | 15.1 | 13.4 | 13.3 | 23.4 | 14.2 | 14.0 | 17.6 | 16.9 | 16.8 | 19.3 | 26.6 | 26.6 | 25.4 |
| South | 37.9 | 38.0 | 35.4 | 39.1 | 39.0 | 37.2 | 26.9 | 27.0 | 26.9 | 25.1 | 25.1 | 22.4 | 62.2 | 62.7 | 51.8 | 32.2 | 32.8 | 23.6 | 34.7 | 34.8 | 32.7 |
| West | 23.8 | 23.8 | 24.7 | 39.1 | 39.4 | 35.9 | 53.2 | 53.2 | 52.6 | 40.3 | 40.3 | 41.8 | 8.8 | 8.8 | 9.6 | 42.1 | 41.7 | 48.7 | 20.0 | 19.8 | 23.5 |
| **Health Behaviors** |  | | |  | | |  | | |  | | |  | | |  | | |  | | |
| Smoking status |  | | |  | | |  | | |  | | |  | | |  | | |  | | |
| Never/quit>12 months prior | 87.3 | 87.8 | 73.9 | 90.7 | 91.0 | 82.9 | 75.7 | 74.8 | 93.5 | 94.5 | 94.7 | 83.1 | 84.2 | 84.7 | 74.0 | 85.2 | 86.1 | 72.3 | 86.2 | 86.9 | 70.1 |
| Former/quit≤12 months ago | 1.2 | 1.2 | 2.4 | 1.1 | 1.0 | 4.0 | 5.9 | 6.2 | 0.0 | 0.7 | 0.8 | 0.0 | 1.4 | 1.4 | 1.2 | 2.8 | 2.6 | 4.5 | 1.2 | 1.1 | 2.5 |
| Current | 11.5 | 11.1 | 23.7 | 8.3 | 8.0 | 13.1 | 18.5 | 19.1 | 6.5 | 4.8 | 4.6 | 16.9 | 14.4 | 13.9 | 24.8 | 12.1 | 11.3 | 23.1 | 12.6 | 12.0 | 27.4 |
| Alcohol consumption |  | | |  | | |  | | |  | | |  | | |  | | |  | | |
| Current (≥1 drink past year) | 69.9 | 70.2 | 64.4 | 62.6 | 62.9 | 53.7 | 50.3 | 51.0 | 36.9 | 52.6 | 52.8 | 54.3 | 63.3 | 63.1 | 68.2 | 69.6 | 69.4 | 74.4 | 74.7 | 75.1 | 66.7 |
| Former (no drinks past year) | 17.0 | 16.7 | 23.8 | 17.9 | 17.7 | 24.1 | 33.8 | 32.3 | 63.1 | 13.3 | 13.4 | 10.9 | 18.9 | 18.9 | 19.3 | 19.3 | 19.2 | 18.3 | 16.7 | 16.3 | 24.6 |
| Lifetime abstinence (<12 drinks in life) | 13.0 | 13.1 | 11.7 | 19.6 | 19.4 | 22.1 | 15.9 | 16.7 | 0.0 | 34.1 | 33.9 | 34.8 | 17.7 | 18.0 | 12.5 | 11.1 | 11.4 | 7.3 | 8.6 | 8.6 | 8.7 |
| Leisure-time physical activity ^c^ |  | | |  | | |  | | |  | | |  | | |  | | |  | | |
| Inactive | 26.5 | 25.8 | 44.3 | 35.3 | 34.7 | 51.8 | 30.3 | 29.1 | 57.7 | 24.0 | 23.5 | 37.6 | 31.7 | 31.2 | 41.8 | 29.2 | 28.3 | 43.6 | 23.5 | 22.6 | 43.6 |
| Insufficiently active | 25.6 | 25.7 | 23.4 | 24.9 | 25.1 | 18.4 | 29.0 | 29.9 | 11.0 | 28.9 | 28.9 | 30.8 | 26.3 | 26.8 | 16.9 | 23.2 | 23.4 | 20.2 | 25.3 | 25.2 | 26.2 |
| Sufficiently active | 47.8 | 48.5 | 32.3 | 39.8 | 40.2 | 29.7 | 40.7 | 41.0 | 31.4 | 47.1 | 47.6 | 31.6 | 42.0 | 42.0 | 41.3 | 47.6 | 48.4 | 36.2 | 51.3 | 52.2 | 30.2 |
| Usual sleep duration |  | | |  | | |  | | |  | | |  | | |  | | |  | | |
| Short (<7 hours) | 30.3 | 29.7 | 45.4 | 31.1 | 30.4 | 52.2 | 37.0 | 35.5 | 65.9 | 27.6 | 27.5 | 43.1 | 39.3 | 38.4 | 57.9 | 38.1 | 37.3 | 51.5 | 28.4 | 27.9 | 40.2 |
| Recommended (≥7 hours) | 69.7 | 70.3 | 54.6 | 68.9 | 69.6 | 47.8 | 63.0 | 64.5 | 34.1 | 72.4 | 72.5 | 56.9 | 60.7 | 61.6 | 42.1 | 61.9 | 62.7 | 48.5 | 71.6 | 72.1 | 59.8 |
| Infrequent Insomnia symptoms (yes) ^d^ | 76.7 | 77.8 | 49.6 | 81.7 | 83.1 | 42.0 | 66.4 | 67.8 | 37.5 | 87.3 | 87.7 | 62.3 | 79.4 | 80.5 | 57.4 | 72.3 | 74.2 | 45.4 | 74.3 | 75.3 | 50.4 |
| Restorative sleep (yes) ^e^ | 57.0 | 58.3 | 25.7 | 55.8 | 57.0 | 22.1 | 55.4 | 56.1 | 39.9 | 63.5 | 64.0 | 32.7 | 54.4 | 55.6 | 29.3 | 53.5 | 54.3 | 38.2 | 57.0 | 58.5 | 24.5 |
| **Clinical Characteristics** |  | | |  | | |  | | |  | | |  | | |  | | |  | | |
| Ever had depression (yes) ^f^ | 18.5 | 16.9 | 55.2 | 14.0 | 12.6 | 55.9 | 23.5 | 22.3 | 49.3 | 7.4 | 6.7 | 46.5 | 14.3 | 12.7 | 46.5 | 18.5 | 17.0 | 40.7 | 21.7 | 20.1 | 58.8 |
| Body mass index category |  | | |  | | |  | | |  | | |  | | |  | | |  | | |
| Underweight (<18.5 kg/m^2^) | 1.7 | 1.6 | 2.8 | 1.0 | 0.9 | 2.1 | 2.5 | 2.7 | 0.0 | 3.5 | 3.6 | 0.0 | 1.3 | 1.4 | 0.0 | 2.5 | 2.7 | 0.0 | 1.7 | 1.6 | 3.9 |
| Recommended (18.5-<25 kg/m^2^) | 31.2 | 31.3 | 28.2 | 23.7 | 23.8 | 19.2 | 29.1 | 27.6 | 56.2 | 54.1 | 54.4 | 27.6 | 23.4 | 23.1 | 30.0 | 34.5 | 34.9 | 26.0 | 32.6 | 32.8 | 28.9 |
| Overweight (25-<30 kg/m^2^) | 33.7 | 34.0 | 27.0 | 37.5 | 37.7 | 33.5 | 31.4 | 31.8 | 24.4 | 30.5 | 30.4 | 43.7 | 32.2 | 32.7 | 22.3 | 28.4 | 27.9 | 38.4 | 33.5 | 33.8 | 26.3 |
| Obesity (≥30 kg/m^2^) | 33.4 | 33.0 | 42.0 | 37.7 | 37.5 | 45.2 | 37.0 | 37.9 | 19.4 | 11.9 | 11.5 | 28.7 | 43.0 | 42.8 | 47.7 | 34.6 | 34.5 | 35.6 | 32.2 | 31.8 | 40.9 |
| General health status |  | | |  | | |  | | |  | | |  | | |  | | |  | | |
| Fair/poor | 13.9 | 12.3 | 51.8 | 18.3 | 17.0 | 58.2 | 20.9 | 19.2 | 58.1 | 11.0 | 10.2 | 52.0 | 19.4 | 17.6 | 54.5 | 18.1 | 16.1 | 48.4 | 12.4 | 10.6 | 51.1 |
| Good/very good/excellent | 86.1 | 87.7 | 48.2 | 81.7 | 83.0 | 41.8 | 79.1 | 80.8 | 41.9 | 89.0 | 89.8 | 48.0 | 80.6 | 82.4 | 45.5 | 81.9 | 83.9 | 51.6 | 87.6 | 89.4 | 48.9 |
| Abbreviations: SE (standard error), NH (non-Hispanic) | | | | | | | | | | | | | | | | | | | | | |
| Note: Data are presented as column percentages or means and standard errors. Percentages may not sum to 100 due to missing rounding. All estimates are weighted for the survey's complex sampling design. All estimates are age-standardized to theU.S.2020 population, except for age. | | | | | | | | | | | | | | | | | | | | | |
| ^a^ NH-Other single and multiple races and ‘Other group’ is defined as persons identifying with racial groups not explicitly listed in the standard categories.  ^b^ Life satisfaction was ascertained based on the questions, 'In general, how satisfied are you with your life? Are you very satisfied, satisfied, dissatisfied, or very dissatisfied?'. Life satisfaction was dichotomized as yes (a response of 'satisfied' or 'very satisfied') vs. no (a response of 'very dissatisfied' or 'dissatisfied'). | | | | | | | | | | | | | | | | | | | | | |
| ^c^ Leisure-time physical activity was defined using the 2018 Health and Human Services Physical Activity guidelines which state “recommend that adults complete at least 150 minutes to 300 minutes of moderate-intensity activity, or 75 minutes to 150 minutes of vigorous-intensity aerobic activity per week, as well as moderate or greater intensity muscle strengthening activities on two or more days a week."  ^d^ Infrequent Insomnia symptoms (yes) defined as difficulty falling or staying asleep never/some days. | | | | | | | | | | | | | | | | | | | | | |
| ^e^ Restorative sleep (yes) defined as most days/every day waking up feeling rested in the past 30 days. | | | | | | | | | | | | | | | | | | | | | |
| ^f^ Depression was defined by the 2019 Field Representative's Manual as a major depressive disorder or as clinical depression that is a common but serious mood disorder. It causes severe symptoms that affect how you feel, think, and handle daily activities, such as sleeping, eating, or working.” | | | | | | | | | | | | | | | | | | | | | |

**Supplemental Figure 2. Prevalence of life satisfaction by race and ethnicity and age, National Health Interview Survey, 2022, (N=25,090)**

Abbreviations: NH (non-Hispanic). Note: Data are presented as column percentages. Percentages may not sum to 100 due to missing rounding. All estimates are weighted for the survey's complex sampling design. All estimates are age-standardized to the U.S. 2020 population, except for age.

NH-Other single and multiple races and ‘Other group’ is defined as persons identifying with racial groups not explicitly listed in the standard categories.

Life satisfaction was ascertained based on the questions, 'In general, how satisfied are you with your life? Are you very satisfied, satisfied, dissatisfied, or very dissatisfied?'. Life satisfaction was dichotomized as yes (a response of 'satisfied' or 'very satisfied') vs. no (a response of 'very dissatisfied' or 'dissatisfied').

**Supplemental Figure 3. Prevalence of life satisfaction by race and ethnicity and sex, National Health Interview Survey, 2022, (N=25,090)**

Abbreviations: NH (non-Hispanic)

Note: Data are presented as column percentages or means and standard errors. Percentages may not sum to 100 due to missing rounding. All estimates are weighted for the survey's complex sampling design. All estimates are age-standardized to the U.S. 2020 population, except for age.

NH-Other single and multiple races and ‘Other group’ is defined as persons identifying with racial groups not explicitly listed in the standard categories.

Life satisfaction was ascertained based on the questions, 'In general, how satisfied are you with your life? Are you very satisfied, satisfied, dissatisfied, or very dissatisfied?'. Life satisfaction was dichotomized as yes (a response of 'satisfied' or 'very satisfied') vs. no (a response of 'very dissatisfied' or 'dissatisfied').
